# Mechanistic insights into metal ion activation and operator recognition by the ferric uptake regulator

**DOI:** 10.1038/ncomms8642

**Published:** 2015-07-02

**Authors:** Zengqin Deng, Qing Wang, Zhao Liu, Manfeng Zhang, Ana Carolina Dantas Machado, Tsu-Pei Chiu, Chong Feng, Qi Zhang, Lin Yu, Lei Qi, Jiangge Zheng, Xu Wang, XinMei Huo, Xiaoxuan Qi, Xiaorong Li, Wei Wu, Remo Rohs, Ying Li, Zhongzhou Chen

**Affiliations:** 1 State Key Laboratory of Agrobiotechnology, China Agricultural University, Beijing 100193, China; 2 Experimental Research Center, China Academy of Chinese Medical Sciences, Beijing 100700, China; 3 Molecular and Computational Biology Program, Departments of Biological Sciences, Chemistry, Physics, and Computer Science, University of Southern California, Los Angeles, California 90089, USA; 4 Institute of Apicultural Research, Key Laboratory of Pollinating Insect Biology, Chinese Academy of Agricultural Science, Beijing 100093, China

## Abstract

Ferric uptake regulator (Fur) plays a key role in the iron homeostasis of prokaryotes, such as bacterial pathogens, but the molecular mechanisms and structural basis of Fur–DNA binding remain incompletely understood. Here, we report high-resolution structures of *Magnetospirillum gryphiswaldense* MSR-1 Fur in four different states: apo-Fur, holo-Fur, the Fur–*feoAB1* operator complex and the Fur–*Pseudomonas aeruginosa* Fur box complex. Apo-Fur is a transition metal ion-independent dimer whose binding induces profound conformational changes and confers DNA-binding ability. Structural characterization, mutagenesis, biochemistry and *in vivo* data reveal that Fur recognizes DNA by using a combination of base readout through direct contacts in the major groove and shape readout through recognition of the minor-groove electrostatic potential by lysine. The resulting conformational plasticity enables Fur binding to diverse substrates. Our results provide insights into metal ion activation and substrate recognition by Fur that suggest pathways to engineer magnetotactic bacteria and antipathogenic drugs.

## Abstract (web summary)

The regulation of iron levels is an important physiological process as excess cellular iron is highly toxic. Here the authors present several structures of a bacterial ferric uptake regulator (Fur) in complex with the Fe2^+^ transport protein operator and Fur box, shedding light on how iron promotes DNA recognition by Fur.

## Graphical abstract

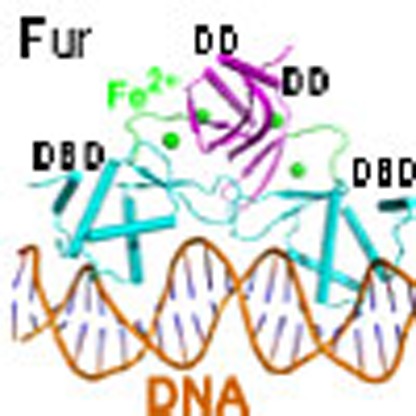

Iron is essential for many biological processes in almost all living organisms^1^,^2^. However, high concentrations of Fe(II) are toxic due to the formation of highly reactive radicals via the Fenton reaction^3^. To survive, the cell has strategies for tightly regulating the cytoplasmic iron level. In most bacteria, iron homeostasis is regulated primarily by the ferric uptake regulator (Fur). Exceptions to Fur use in bacteria include Gram-positive bacteria with high genomic GC content, such as *Corynebacterium* and *Streptomyces*, in which the diphtheria toxin repressor (DtxR) regulates iron homeostasis^4^,^5^.

Fur, which was first discovered in *Escherichia coli*, is a global transcriptional regulator that directly controls the transcription of over 90 genes involved in iron uptake, storage and metabolism^6^. Fur controls the iron-regulated expression of Shiga toxin in *Shigella dysenteriae*, haemolysin in *E. coli* and exotoxin A in *Pseudomonas aeruginosa*^7^,^8^. Fur is vital to host/parasite interactions because it controls the expression of many proteins that function in iron-scavenging and uptake systems that can acquire iron directly from haem or specifically internalize host iron-binding proteins^9^. Analyses of different Fur-regulated promoters in *E. coli* and *P. aeruginosa* led to identification of the consensus Fe(II)-Fur-binding sequence, 5′-GATAATGATAATCATTATC-3′, known as the ‘Fur box' (Supplementary Table 1)^10^,^11^. In the classic Fur regulation pattern, monomeric Fur binds iron, dimerizes and then binds to the promoter of Fur-regulated genes to occlude binding of RNA polymerase and repress gene transcription^12^. In addition to Fe(II), Fur is activated by other divalent transition metal ions, with the following order of activation: Zn(II)≫Co(II)>Fe(II)>Mn(II)^13^.

Several holo-Fur structures have been solved, revealing a modular domain organization including an N-terminal DNA-binding domain (DBD) and a C-terminal dimerization domain (DD). Metal ions mediate the binding of Fur to operators, and metal ion-binding sites are diverse in bacterial species^7^,^14^,^15^,^16^,^17^. Recently, a minor-groove readout mechanism used by Fur has been proposed^18^. However, the molecular mechanisms for metal ion activation and operator recognition by Fur remained poorly understood. Unresolved issues include the mechanisms by which metal ions activate Fur, and explanations for why Fur has such a broad substrate-binding ability. Moreover, the lack of Fur–DNA complex structures has prevented the design of antipathogenic drugs.

A recent study identified a *fur* gene in *Magnetospirillum gryphiswaldense* MSR-1. This study demonstrated that *fur* can directly regulate the expression of several key genes involved in iron transport and oxygen metabolism, and that *fur* can complement a *fur*-defective mutant of *E. coli* in an iron-responsive manner *in vivo*^19^,^20^. The *fur* gene plays a key role in the formation of magnetosomes, which are uniform, nanosized and membrane-enclosed magnetic crystals that have been used in many biomedical applications due to their unique features^21^.

In this study, we present six crystal structures of apo-Fur, holo-Fur, Fur in complex with the Fe^2+^ transport protein (*feoAB1*) operator and the *P. aeruginosa* (or *E. coli*) Fur box. To address the roles of each metal- and DNA-binding site, we performed biochemical, biophysical and *in vivo* analyses of Fur wild type (WT) and mutants. These structures, in conjunction with mutagenesis and functional studies, allow us to uncover the possible mechanisms of the metal ion-induced conformational changes and the DNA recognition of broad target genes by Fur.

## Results

### Fur recognizes the *feoAB1* operator and *P. aeruginosa* Fur box

**Figure 1:**
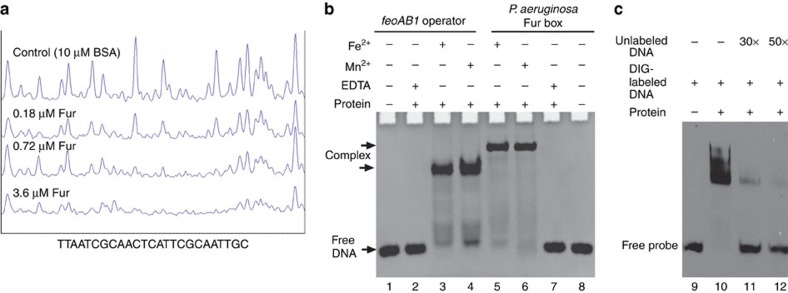
MgFur specifically binds to the *feoAB1* operator and the *P. aeruginosa* Fur box. (**a**) DNase I footprinting of the coding strand of the *feoAB1* promoter region identified using MgFur. Fluorograms correspond to the control protein (10 μM bovine serum albumin) and to the protection reaction with increasing concentrations of Fur (0.18, 0.72 and 3.6 μM). The corresponding protected sequence is shown below. (**b**) Binding of Fur to the *feoAB1* operator or the *P. aeruginosa* Fur box depends on metal ions. (**c**) 3′ DIG-labelled DNA probe and Fur protein were incubated with varying amounts of unlabelled DNA. The DNA is that of the *feoAB1* operator, and the position of the free probe is indicated. Lane 9, DNA probe; lane 10, standard binding reaction; lane 11, standard binding reaction+30-fold molar excess of unlabelled probe DNA; lane 12, standard binding reaction+50-fold molar excess unlabelled DNA.

MSR-1 Fur (MgFur) interacts with the *feoAB1* promoter^19^. To determine the specific Fur-binding sequences, DNase I footprinting of the *feoAB1* promoter was performed in the presence of manganese ions. The *feoAB1*-coding strand showed a 25-base pair (bp) main protected region with the sequence 5′-TTAATCGCAACTCATTCGCAATTGC-3′, referred to as the ‘*feoAB1* operator' (Fig. 1a). The operator did not have the typical features of the Fur box with three adjacent 5′-GATAAT-3′ hexamers. Additional gel shift assays showed that Fur binds specifically to the *feoAB1* operator and the *P. aeruginosa* Fur box. When EDTA was added to chelate metal ions, Fur lost its DNA-binding ability and was unable to bind to the *feoAB1* operator or the *P. aeruginosa* Fur box (Fig. 1b). Competitive binding of excess free DNA to Fur protein confirmed the interaction between holo-Fur and the *feoAB1* operator (Fig. 1c).

### Apo-Fur forms a transition metal ion-independent dimer

**Figure 2:**
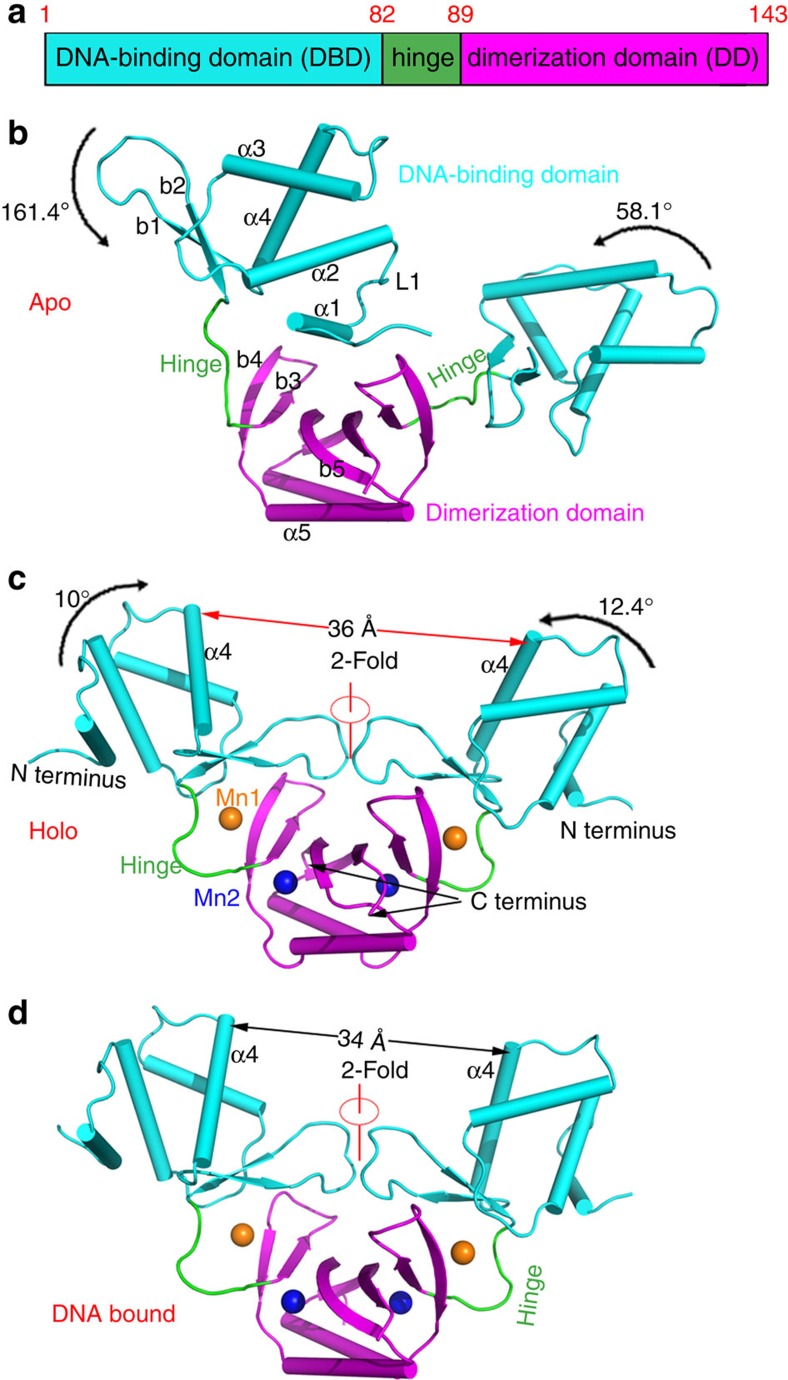
Conformational flexibility of MgFur. (**a**) Domain organization of Fur: N-terminal DBD (cyan), hinge (green) and C-terminal DD (magenta). Tube representation of (**b**) MSR-1 apo-Fur, (**c**) manganese-activated (holo-Fur) and (**d**) operator-bound Fur dimer. Colour code is the same as in **a**. The bound metal ions Mn1 and Mn2 are labelled. The conformations of the two monomers in apo-Fur differ greatly, whereas the two monomers in holo-Fur and in the DNA-bound complex are related by a twofold rotation axis.

The apo-MgFur structure was determined at 1.55 Å resolution (Table 1). The results showed that apo-Fur is made of two monomers that form a stable dimer (Fig. 2a,b), with both monomers consisting of residues 1–134 and nine disordered C-terminal residues. Each monomer exhibited a modular architecture, including an N-terminal DBD and C-terminal DD, connected by a flexible hinge formed by residues 83–88 (Supplementary Fig. 1a). The DBD was composed of four consecutive α-helices (α1, α2, α3 and α4) followed by an antiparallel β-sheet (β1−β2) (Fig. 2b). The DD contained a mixed-α/β domain, in which α5 intersected between β4 and β5 (Supplementary Fig. 1a). The dimeric interface was primarily formed by the DD, which was composed of two α5 and two β5 that formed intermolecular hydrophobic interactions and an antiparallel β-sheet, respectively (Supplementary Fig. 2a,b). The buried interface comprised 1,628.4 Å^2^ per molecule, indicating a very strong interaction between the monomers. Additional size-exclusion chromatography and analytical ultracentrifugation results confirmed that apo-Fur exists as a dimer in solution (Supplementary Fig. 2c,d).

Overall conformations of the two Fur monomers, especially in the hinge region, differed greatly, with an overall root mean squared deviation (r.m.s.d.) of 4.6 Å (Fig. 2b; Supplementary Fig. 3a; Supplementary Movie 1). However, their DD and DBD conformations were almost identical (r.m.s.d.=0.9 and 0.4 Å, respectively). Analysis of the crystal packing of the apo-Fur structure revealed that the DBD is stabilized by other DBDs and DDs from symmetric units. As apo-Fur is mostly dimeric in solution, the positions of the DBD and hinge are flexible (Supplementary Movie 1). Close analysis of the calculated Fourier maps in proximity to the metal ion-binding sites failed to detect any additional electron density. No absorption peak of transition metal ions was found by X-ray absorption spectroscopy, suggesting that the metal ion-binding sites are not occupied in apo-Fur.

### Metal ion binding induces conformational changes of the DBD

Despite testing different metal ions through extensive screening, we were unable to obtain crystals for holo-Fur. Sequence alignment showed that MgFur has a unique motif, made of Cys9, Met14 and Met16 (Supplementary Fig. 1a). Therefore, we hypothesized that these three residues might be easily oxidized during protein crystallization.

We produced a C9L/M14L/M16V-triple mutant that successfully generated diffraction-quality holo-Fur crystals. The overall structure of holo-Fur was a homodimer with the same secondary structure as that of apo-Fur (Fig. 2b,c). The two monomers in holo-Fur had nearly identical conformations (r.m.s.d.=0.36 Å) that differed substantially from those in apo-Fur. The overall holo-Fur structure from MSR-1 was similar to that from other prokaryotes (for example, r.m.s.d.=1.9 Å compared with that from *Vibrio cholerae*)^15^. The large difference between the apo- and holo-Fur structures arose from the conformations of the two N-terminal DBDs and two hinges. Thus, the binding of two Mn^2+^ ions stabilized the hinge conformation and induced profound conformational changes of the DBD (Supplementary Fig. 3b; Supplementary Movie 1). By binding metal ions, the dimeric DD holds the DBD, and the DD is prepared to bind target DNA (Supplementary Fig. 3c).

**Figure 3:**
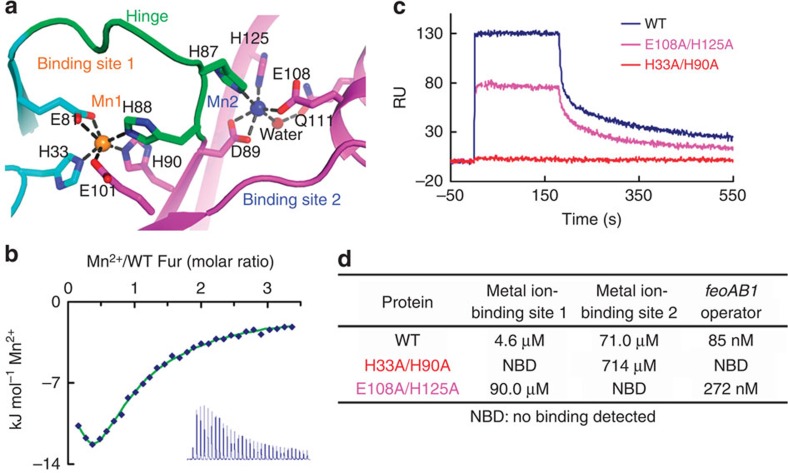
Metal ion-binding sites and binding affinities of Fur mutants. (**a**) Coordination of the metal ion-binding sites S1 and S2. Colour code is the same as in Fig. 2. (**b**) Calorimetric titration of WT Fur (60 μM) with MnCl_2_ (2 mM). Data were fitted to a two-binding-site model. Calorimetric titration of the other Fur mutants at S1 or S2 was also performed and was fitted to the one-binding-site model. (**c**) Binding of WT or mutant Fur protein (2 μM) to the *feoAB1* operator determined by SPR. (**d**) Binding affinities of Fur proteins to metal ion and the *feoAB1* operator determined by ITC and SPR, respectively.

Each holo-Fur monomer contains two Mn^2+^-binding sites (site 1 and site 2). Site 1 links the DBD and DD. This site contains a manganese ion, Mn1, that is hexacoordinated by two residues (H33 and E81) from the DBD and three residues (H88, H90 and E101) from the DD (Fig. 3a). Through using site 1, the DBD is recruited to the DNA-binding site. Site 2 resides almost entirely in the DD, within the β3 and β5 strands and α5 helix. In site 2, the manganese ion, Mn2, is also hexacoordinated by residues H87, D89, E108, H125 and Q111, while a water molecule mediates interactions with the latter (Supplementary Table 2; Supplementary Fig. 3a,e).

### Site 1 is essential for Fur–DNA binding *in vitro*

The relationship of the two metal-binding sites in holo-Fur and their functional roles are still unclear. To address these issues, we performed titrations of the WT protein and two double mutants (site-1 mutant H33A/H90A and site-2 mutant E108A/H125A) with manganese ions by isothermal titration calorimetry (ITC). The titration curve of WT Fur fits a two-binding-site model (Fig. 3b), whereas the mutants only fit a one-binding-site model, implying that one binding site was lost. WT Fur had a high-affinity binding site 1 (*K*_d_=4.6 μM) and a low-affinity binding site 2 (*K*_d_=71.0 μM). The two binding sites exhibited concerted efforts, as disrupting one binding site lowered the binding affinity of the other site by about one order of magnitude (Fig. 3d).

We examined whether these binding sites are functionally relevant to the DNA-binding affinity through surface plasmon resonance (SPR) experiments. The strongest DNA binding was observed for the WT protein (*K*_*D*_=85 nM). The E108A/H125A mutation substantially decreased the affinity (*K*_*D*_=272 nM), and the H33A/H90A mutation abolished binding (Fig. 3c,d; Supplementary Fig. 4c,d). These results demonstrate that the metal ion-binding site 1 is sufficient for Fur to gain detectable DNA-binding affinity *in vitro*. Although site 2 was not essential for DNA binding, its disruption substantially reduced the DNA-binding ability.

### Overall structure of Fur-Mn^2+^–DNA ternary complex

Fur specifically recognizes the target sequences of regulated genes and affects their transcription through poorly understood mechanisms. We solved the structures of Fur bound to 25-bp oligonucleotides containing either the *feoAB1* operator or the *P. aeruginosa* Fur box. We first tried to co-crystallize MgFur with different lengths of the *feoAB1* operator containing the specific sequence; however, all of the crystals diffracted poorly. To obtain diffraction-quality crystals, we mutated the *feoAB1* operator to generate a near-perfect inverted repeat. The sequence in the co-crystal of the Fur-Mn^2+^–*feoAB1* operator ternary complex was 5′-TTAATTGCAAATCATTTGCAATTGC-3′, carrying three single-base-pair mutations (underlined). MgFur bound to this sequence and to the original sequence with similar binding affinities (Supplementary Fig. 4c,f). We also screened complexes of the Fur–*P. aeruginosa* Fur box with different sequences at the end regions. The sequence in the final co-crystal of the Fur-Mn^2+^–*P. aeruginosa* Fur box complex was 5′-CGCGATAATGATAATCATTATCCGC-3′. The functional structure was generated by a crystallographic twofold axis.

**Figure 4:**
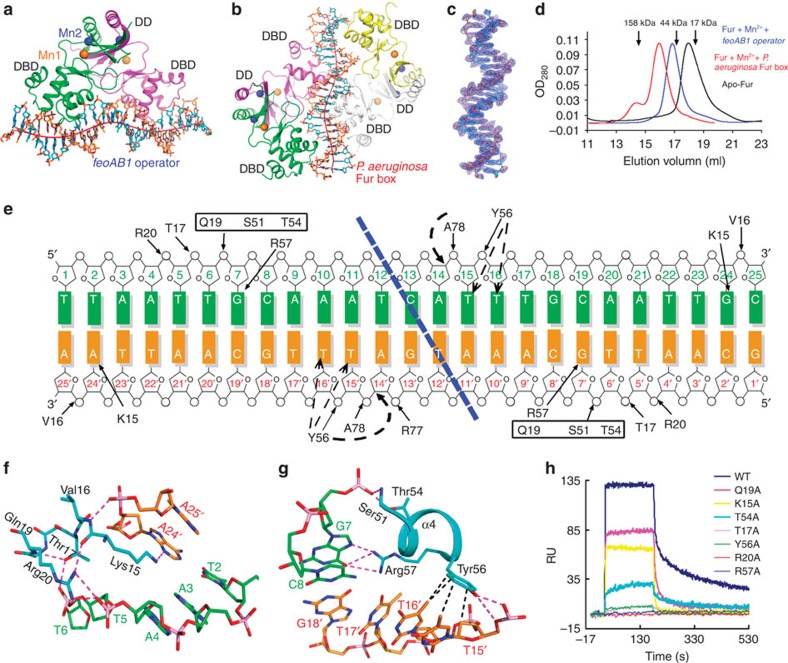
Structures of the Fur-Mn^2+^–DNA ternary complex. Overall structures of the *feoAB1* operator in complex with two Fur monomers (**a**) and the *P. aeruginosa* Fur box in complex with four Fur monomers (**b**). Fur is coloured by subunit. The DNA helical axis calculated with CURVES (see Methods) is shown as a red tube. Fur conformations are almost the same in these complex structures. (**c**) 2*F*_o_*−F*_c_ electron density map (1.0 *σ*) of the bound *P. aeruginosa* Fur box. (**d**) Analytical gel filtration profiles of apo-Fur, and Fur in complex with different DNA fragments in the presence of Mn^2+^. Elution volumes of the molecular mass standards are marked at the top of the panel. (**e**) Schematic diagram summarizing the Fur–*feoAB1* operator interactions. Solid and dashed arrows indicate hydrogen bonding and van der Waals interactions, respectively. Dashed blue line indicates the boundary between nucleic acids bound by the two monomers. A close-up view of the interactions of loop 1 (L1) with the minor groove (**f**) and contacts between the α4 helix and the major groove (**g**). Dashed magenta lines indicate hydrogen bonding, and dashed black lines indicate van der Waals interactions. (**h**) Overlay plot of subtracted sensorgrams generated with binding of WT or mutant Fur protein (2 μM) to the *feoAB1* operator determined by SPR. K15A and Y56A mutants showed weak binding, whereas no binding of R57A mutants was detected.

The solved structure of the Fur-Mn^2+^–*feoAB1* operator complex contained one Fur dimer bound to double-stranded DNA (dsDNA) (Fig. 4a). Surprisingly, binding of two Fur dimers to one dsDNA target site was found in the Fur-Mn^2+^–*P. aeruginosa* Fur box structure, with one dimer bound close to the 5′ end of the coding strand and the other dimer close to the 3′ end (Fig. 4b). The electron density corresponding to the *P. aeruginosa* Fur box was sufficiently well resolved to allow unambiguous assignment of the DNA nucleotide sequence, except for one missing base at each 3′ end (Fig. 4c; Supplementary Fig. 5a,b). Consistent with this observation, each complex was eluted as a single peak from a size-exclusion column with a molecular weight corresponding to that of the complex structure (Fig. 4d). Combined with the findings from the gel shift assay (Fig. 1b, lanes 3–6) and a previous report^22^, these results suggest that Fur can bind DNA targets at different ratios.

Superimposition of all DNA-bound Fur subunits with the holo-Fur subunits using all Cα atoms revealed moderate differences (r.m.s.d.=1.2–2.1 Å). In both complexes, DNA binding resulted in a decreased distance (from 36 to 34 Å) between the α4 helices of the two monomers (Fig. 2d; Supplementary Fig. 3c), such that the α4 recognition helices fit better into consecutive major groove regions.

### Fur recognizes DNA using base and shape readout modes

Fur-bound DNA molecules were in a B-form conformation and slightly bent away from the α4 helix at the interface with the protein (Fig. 4a,b). Despite the sequence variation between the two DNA target sites, each Fur monomer formed similar contacts with both dsDNA strands using its DBD. The DBDs in one Fur dimer did not interact with each other. The DBD recognized a characteristic 10- to 11-bp DNA target, which contained an important G base, a conserved T base and an AT-rich region that was characterized by a narrow minor groove (Fig. 4e; Supplementary Fig. 5a,b). Most interactions were with residues 15–20 in the L1 loop (Fig. 4f; Supplementary Fig. 5c) and with the α4 recognition helix (Fig. 4g; Supplementary Fig. 5d), which interacted with functional groups at the major groove edges of the base pairs forming the binding site (base readout)^23^. The L1 loop formed contacts with DNA in the minor groove (shape readout)^24^. This recognition mechanism differs substantially from that of the iron-responsive transcriptional regulator DtxR, in which a β-sheet (forming a ‘wing') is used to bind DNA in the minor groove^25^ (Supplementary Fig. 3d).

Most interactions between Fur and DNA occurred on the phosphodiester backbone. Side chains of Thr17, Gln19 and Arg20 and the backbone of Val16 in the L1 loop formed hydrogen bonds or electrostatic interactions with DNA phosphate groups in the minor groove. Another sequence-independent interaction with the DNA backbone occurred via the side chains of Thr54 and Tyr56 and the backbone of Ser51 and Ala78 (Fig. 4e–g; Supplementary Fig. 5a–d).

Lys15, Tyr56 and Arg57 recognized DNA through three different modes of interaction (Supplementary Fig. 5a). In the first recognition mode, the phenyl ring of Tyr56 in the α4 helix forms van der Waals interactions with one or two consecutive T bases in the major groove, such as the methyl groups of T15′ and T16′ of the *feoAB1* operator or T12′ of the *P. aeruginosa* Fur box (Fig. 4g; Supplementary Fig. 5d). This observation is consistent with our SPR finding of a very weak DNA-binding signal for the Y56A mutant (Fig. 4h), as well as with previous hydroxyl radical footprinting and missing-T assay results demonstrating that loss of thymine nucleotides impairs the DNA binding of Fur^11^. Therefore, the thymine base is an essential recognition element for direct contacts with Fur.

In the second recognition mode, Arg57 inserts into the major groove, forming bidentate hydrogen bonds between its guanidinium group and atoms O6 and N7 of a conserved guanine, such as G7 of the *feoAB1* operator and G10 of the *P. aeruginosa* Fur box (Fig. 4g; Supplementary Fig. 5d). Arg57 also forms one hydrogen bond with T15′ of the *P. aeruginosa* Fur box. The importance of this interaction was confirmed by the SPR experiments, in which no DNA-binding ability was detected for mutant R57A (Fig. 4h).

In the third recognition mode, Lys15 of the L1 loop inserts into the minor groove with few direct interactions with DNA. For example, it forms hydrogen bonds with A24′ of the *feoAB1* operator or with T6 and T21′ of the *P. aeruginosa* Fur box (Fig. 4f; Supplementary Fig. 5c). However, these interactions do not have any base specificity^26^. To test whether Lys15 is required for the DNA-binding ability of Fur, we measured the *K*_*D*_ values for the binding of the K15A mutant to the *feoAB1* operator. The K15A mutant had a substantially lower binding affinity than the WT protein (977 versus 85 nM; Supplementary Fig. 4c,e).

### Lys15 and sites 1 and 2 are essential for Fur function *in vivo*

Both Lys15 and metal ion-binding site 2 affected the DNA-binding ability of Fur and were not indispensable *in vitro* (Supplementary Fig. 4e,h). We analysed the physiological significance of Lys15 and binding site 2 *in vivo* by generating a *fur*-defective strain of MSR-1 (F4) and complementary strains capable of expressing WT (F4C), the single mutants K15A and R57A, binding site-1 mutant H33A/H90A and binding site-2 mutant E108A/H125A. We examined the regulatory effect of Fur and Fur mutants on the *feoAB1* gene involved in iron metabolism.

**Figure 5:**
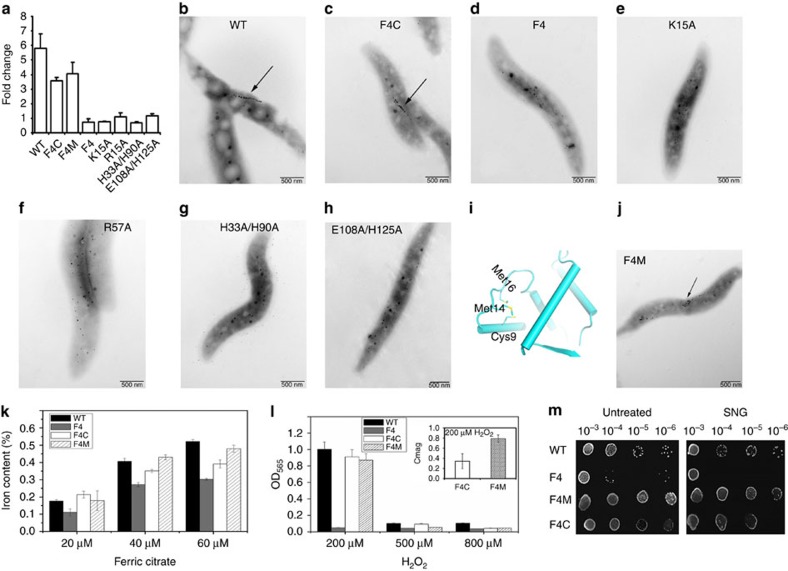
Compromised *in vivo* activities of Fur mutants. (**a**) Comparison of *feoAB1* expression level by cells treated with the iron chelator DIPy (30 μM) or ferric citrate (60 μM). Each column represents the expression fold change between low-iron and high-iron conditions in MSR-1 cells. (**b**–**h**) TEM micrographs of magnetosomes from different Fur mutants. Arrow indicates the magnetosome chain. (**i**) Structure of the sulphur-rich centre in the DBD. (**j**) Cells of sulphur-rich centre mutant F4M were observed by electron microscopy, indicating formation of a normal magnetosome chain. (**k**) Intracellular iron content of each cell under different iron concentrations. Iron concentration in F4M was higher than in F4C cells under 40–60 μM iron. (**l**) H_2_O_2_ tolerance of each strain. Although growth level was almost same between F4C and F4M strains under the 200 μM H_2_O_2_ condition, the magnetosome synthesis ability in F4C cells (Cmag=0.34±0.14) was lower than that in F4M cells (Cmag=0.79±0.07). (**m**) SNG tolerance of each strain. F4M showed the best growth status among the four tested strains, both before and after treatment with SNG. The results indicated that residues Cys9, Met16 and Met19 were related to the oxygen sensitivity of Fur in magnetotactic bacteria. Each assay was performed in three independent replicates, and values shown are means with s.d.

Under the iron-repleted condition, relative expression levels of the *feoAB1* gene detected by quantitative real-time PCR (qPCR; Fig. 5a, left two lanes) in WT and F4C strains were 5.81- and 3.57-fold higher, respectively, than levels under the iron-depleted condition. These findings indicate that the *feoAB1* gene is obviously repressed in the WT and F4C strain. Consistent with the *in vitro* results, in the F4 strain and strains containing R57A and the binding site-1 mutant H33A/H90A, the difference between the expression levels of *feoAB1* under the iron-repleted and iron-depleted conditions was not obvious, suggesting that *feoAB1* gene expression is not subject to regulation. Although the K15A and binding site-2 E108A/H135A mutants still had comparable DNA-binding abilities *in vitro*, both strains lost the ability to repress *feoAB1* gene expression under the iron-repleted condition.

The *fur* gene plays a key role in magnetosome formation by MSR-1 (refs 19, 20). Transmission electron microscopy (TEM) results confirmed that all strains containing the four Fur mutants and the F4 strain substantially reduced the total size of magnetosomes that formed, with the smaller magnetosomes being dispersed in cells and arranged in irregular chains (Fig. 5d–h). Magnetosomes of the WT and F4C strains did not show this reduction in size (Fig. 5b,c). These results suggest that Lys15 and the two binding sites are essential for Fur function *in vivo*.

### Lys15 recognizes DNA through minor-groove shape readout

**Figure 6:**
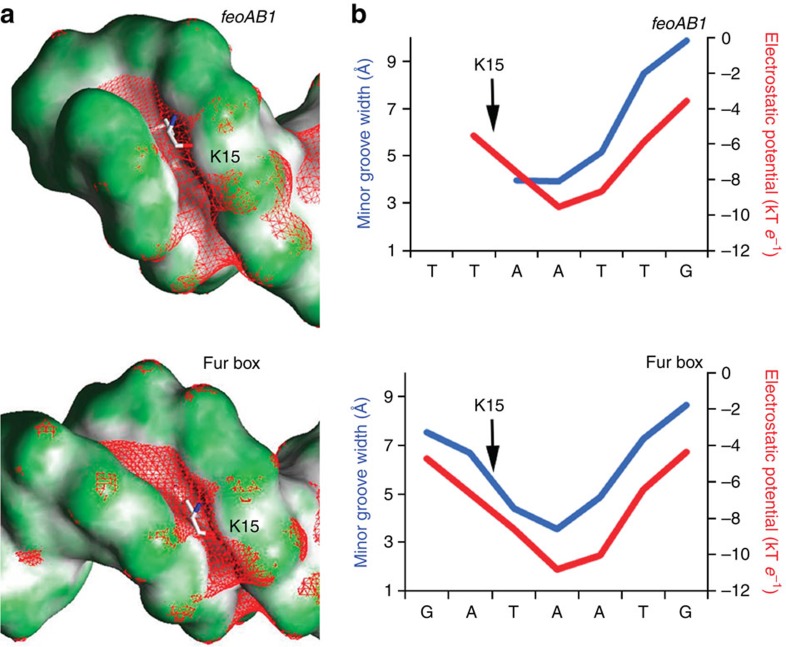
Narrow minor-groove geometry of DNA where the Lys15 residue binds. (**a**) *feoAB1* operator and (**b**) *P. aeruginosa* Fur box. Shape of the molecular surface is shown with GRASP2 (concave surfaces in dark grey, convex surfaces in green^56^). The red mesh represents an isopotential surface at −5 kT e^−1^, calculated with DelPhi at a physiologic ionic strength of 0.145 M (ref. 57). Lys15 residues intrude into the minor groove in a region with enhanced negative electrostatic potential as a result of narrowing the width of the minor groove. The minor-groove width of bound DNA in our crystal structure (blue) and the electrostatic potential in the minor groove calculated with DelPhi (red) illustrate minor-groove-binding sites for the Lys15 residues. The enhanced negative electrostatic potential in the narrower groove region attracts Lys15 residues through favourable electrostatic interactions, a mechanism known as DNA shape readout.

The aforementioned results of two *in vivo* experiments revealed that Lys15 is essential for Fur function. However, structure analysis showed that the Lys15 residues anchored in the minor groove of the Fur-Mn^2+^–*feoAB1* operator and the Fur-Mn^2+^–*P. aeruginosa* Fur box without base-specific contacts. To uncover the recognition mechanism of Lys15, we further analysed the DNA structure. The minor-groove region bound by the Lys15 side chains was narrower than its adjacent regions (Fig. 6, blue line), with a minimum width of about 4 Å (versus 5.8 Å for standard B-DNA). The narrower minor groove might be an intrinsic structural feature of the DNA sequence, or it may be induced by Fur binding.

To distinguish between these possibilities, we probed minor-groove topographies of unbound Fur targets using hydroxyl radical-cleavage intensities^27^. This analysis indicated that both DNA regions of the Lys15 contacts were characterized by an intrinsically narrow groove in the absence of the protein (Supplementary Fig. 6). The negative electrostatic potential in the minor groove is enhanced as the groove width decreases^24^. The electrostatic potential of the narrow minor-groove regions where the Lys15 residues bind were about 3 kT *e*^−1^ more negative than those of the wider minor groove of adjacent regions (Fig. 6, red line). Thus, the positively charged Lys15 residues favourably bind to the intrinsically narrow minor groove with enhanced negative electrostatic potential.

This observation suggests that the binding of Lys15 residues to the target DNA is a specific form of the DNA shape readout mechanism. Lysine, despite its positive charge and abundance on protein surfaces has not been widely associated with this readout mode^24^. Nevertheless, the results in Fur demonstrate that lysine can play this role (Fig. 6). A survey of structures from other protein families revealed additional examples where a lysine binds to narrow minor-groove regions with enhanced negative electrostatic potential (Supplementary Fig. 7).

## Discussion

The classical iron-bound Fur repression model proposes that the Fe^2+^ cofactor binds to apo-Fur monomers, followed by dimerization of the iron-bound monomers^28^. However, prior to this study, no apo-Fur structures without transition metal ions had been reported. All available Fur structures^7^,^14^,^15^,^16^,^17^ possess at least one metal ion per monomer and are dimers. In this study, we solved the first apo-Fur structure without transition metal ions and found that apo-MgFur is a transition metal ion-independent dimer, providing a structural basis for further studies of apo-Fur.

Upon binding of Mn^2+^ and DNA to Fur, we observed two structural changes. First, the DBD underwent large conformational changes. Metal ions greatly stabilized the structure. Differential scanning chromatography (DSC) results confirmed that holo-Fur has higher *T*_m_, *t*_onset_ and Δ*H* values compared with apo-Fur. Second, adding DNA triggered a change of the DBD orientation, thereby enabling certain residues to interact with DNA. The distance between the α4 recognition helices decreased to 34 Å, and the helices became better aligned with consecutive major groove regions. Together, the metal ion-induced caliper-like rotation and movement of the DBDs and the DNA binding account for Fur activation. Overall, the mechanism of Fur regulation differs greatly from that of DtxR^25^,^29^, in which a helix-to-coil transition and no DNA-induced conformational changes were observed.

As a global regulator, Fur recognizes many target sites in the genome^17^,^30^ that differ in length or sequence. Little information is known about the DNA recognition mechanisms employed by Fur. Here, we provide the first co-crystal structures of ternary complexes of Fur family proteins with two DNA targets. The caliper-like DBD employs two types of DNA recognition modes. Recognition of the target DNA by Lys15 occurred through a shape readout mechanism. In agreement with our finding, the minor-groove readout of Hp Fur was previously demonstrated by distamycin competition assays and IC box substitutions^18^. Tyr56 and Arg57 recognized substrates by base readout through hydrophobic interactions and hydrogen bonds, respectively. Sequence alignment and superimposition of the DBDs of other prokaryotic Fur proteins illustrated that these three key DNA-binding residues are highly conserved in almost the same spatial positions (Supplementary Fig. 1c). Consequently, the Fur recognition mechanism revealed in our structures is likely conserved across prokaryotes.

Consensus Fur boxes found in different species further support the above analyses. Other than the aforementioned four base pairs and the AT-rich region, the identity of other base pairs and the order of A/T base pairs in the AT-rich region seem to allow random variations; thus, the sequence recognized by Fur is highly degenerate. Due to the lack of a well-defined sequence specificity, base-pair substitutions are tolerated without overall loss of functionality. Our findings explain why Fur recognizes >90 diverse genes and degenerate sequences in the pathogens *V. cholerae* and *Campylobacter jejuni*^17^,^30^.

We showed that Lys15 and a narrow minor groove are essential features for Fur function. AT-rich sequences tend to form a narrow minor groove because of negative propeller twisting, which is stabilized by inter-base-pair hydrogen bonds in the major groove^31^. We suggest that these sequence-dependent effects on DNA structure confer the specific DNA conformations observed in this study. Consensus sequences bound by Fur in *E. coli*, *P. aeruginosa*, *V. cholerae* and *C. jejuni* are also AT rich^10^,^17^,^30^,^32^. Hydroxyl radical-cleavage intensities demonstrated that the narrow minor groove is an intrinsic feature of the *P. aeruginosa* Fur box and other Fur-binding sequences. In our structures, residues in the L1 loop and α2 helix detected the minor-groove width through interactions with the DNA backbone, and positioned Lys15 so that it inserted into the minor groove and interacted with its negative electrostatic potential. Thus, shape readout, rather than specific sequences for high-affinity recognition, may be a hallmark of the Fur proteins. Instead of being able to read only a stringent promoter sequence, this specific property of recognizing DNA shape confers a global regulatory function to Fur.

Individual contacts between Fur and DNA have precedents in other structural classes of DNA-binding proteins. For example, a recognition helix of a helix-turn-helix motif inserting into the major groove (base readout) is commonly found in repressor proteins. Side chains of positively charged residues inserting into a narrow minor groove are observed in homeodomains and other protein families^24^,^33^. This interplay between base and shape readout has been described for many transcription factors^26^.

However, in contrast to our previous observations for arginine^24^ and histidine^34^, Fur uses lysine to recognize the enhanced negative electrostatic potential in the minor groove. Insertion into the narrow minor groove is associated with desolvation, whose energetic cost is higher for lysine compared with arginine^24^. While lysine insertion into the minor groove has been previously observed^35^, through its recognition of enhanced negative electrostatic potential in narrow minor-groove regions, Fur extends the possible repertoire of shape readout by basic amino acids. A survey of the Protein Data Bank revealed other examples of lysine recognizing the narrow minor groove (Supplementary Fig. 7), although the number of examples was much fewer compared with arginine^24^.

Zinc ions were used in all previous Fur structures^7^,^14^,^15^,^16^,^17^. However, the binding ability and coordination number of Zn^2+^ differ greatly from those of Fe^2+^, and these differences might affect the study results (Supplementary Fig. 3e–g). In contrast, we used Mn^2+^ to achieve reversible binding. Both sites in our structures adopted the typical octahedral geometry of Fe^2+^. Moreover, all of the residues involved in metal ion binding are conserved across Fur proteins from different species. In agreement with the previously reported Mössbauer data of *E. coli* Fur, site 1 is the Fe^2+^-binding site with a 3His/2Glu ligand set^36^. Another study^13^ indicated that *E. coli* Fur has a similar affinity for Mn^2+^ and Fe^2+^. Taken together, these observations suggest that our structures may reflect the physiological state of ferrous ion binding.

Apo-MgFur dimerization is independent of metal ions, and mutations at sites 1 and 2 did not affect dimerization or the secondary structural characteristics. Thus, it can be assumed that both sites play regulatory roles in modulating Fur activity. Our simplified model postulates that Fe^2+^ binding to site 1 activates Fur to bind target DNAs. Without site-1 occupation, Fur cannot bind to or repress any of its target genes; thus, iron homeostasis is inhibited. However, the precise role played by site 2 *in vivo* is currently not understood. One possibility is that, as a global regulator, Fur regulates the expression of diverse genes that may be responsive to different iron concentrations. Site-1 occupation may not be sufficient to guarantee binding to all target genes because of the substantial decrease in DNA-binding affinity after losing site 2. As cellular iron concentrations increase, additional Fe^2+^ binding to site 2 is needed to repress genes that are less sensitive to iron. Hence, sites 1 and 2 can serve as an on–off switch and a fine-tuner, respectively, for activity modulation. This regulatory model is of particular importance for future structure-based studies of inhibitors that target regulator sites.

Our results confirm that Fur can bind to different DNA targets at various ratios. Cooperative binding of several Fur dimers to one Fur box has been reported in other bacteria, such as *Bacillus subtilis*^37^, *Bradyrhizobium japonicum*^38^, *E. coli*^22^, *Helicobacter pylori*^18^ and *Borrelia burgdorferi*^39^, and even in the absence of DNA^40^. Fur-tetramer models have also been proposed^18^,^37^, but no crystal structures were reported. The two Fur dimers are positioned on almost opposite sides of the double helix, in contrast to the positioning of other prokaryotic helix-turn-helix motif class repressors, such as the trp repressor^41^. The phenomenon of two protein dimers binding on almost opposite sides of one dsDNA has been observed in the DtxR–operator complex^25^. However, the mechanisms markedly differ between DtxR and Fur. The two DtxR dimers are arranged so that the dimers do not interact with each other. In contrast, the two Fur dimers do interact, mostly in the DNA major groove and between the two antiparallel α3 helices of the Fur dimers. There are two hydrogen bonds, two water-mediated hydrogen bonds and two electrostatic interactions at the interface between Glu37, Tyr40, Arg41 and Thr44 of each dimer, suggesting that the inferred cooperativity arises from cross-dimer protein–protein contacts. The negative electrostatic potential in the minor groove of the *P. aeruginosa* Fur box is enhanced compared with that of the *feoAB1* operator, which might further increase the binding ability. Thus, cooperativity can explain why the *P. aeruginosa* Fur box can tolerate the loss of shape readout by Lys15 when a Fur monomer is positioned at the end of a DNA fragment in the crystal structure.

The sulphur-rich centre containing Cys9, Met14 and Met16 (Fig. 5i) is unique to MgFur and not found in non-magnetotactic bacteria. These three residues are conserved in magnetotactic bacteria (Supplementary Fig. 1b). Successful creation of diffraction-quality crystals by using the C9L/M14L/M16V-triple mutant suggested that these three residues may be related to oxygen sensitivity. Superimposition of the selenomethionine-substituted and triple-mutated Fur-Mn^2+^–*feoAB1* operators revealed little difference in their structures (r.m.s.d.=0.46 Å).

To confirm the oxygen sensitivity of the sulphur-rich centre, we constructed a complementary strain of *fur*, F4M, in which we replaced the three residues (C9L/M14L/M16V). Compared with F4C cells, the F4M cells not only synthesized normal magnetosome chains, but also facilitated iron uptake and enhanced H_2_O_2_ and streptonigrin (SNG) tolerance (Fig. 5j–m). Although the growth level with 200 μM H_2_O_2_ was almost the same between the two strains, the average magnetic response (Cmag) of the F4M cell suspension (0.79±0.07) was increased 130% compared with that of F4C (0.34±0.14). Thus, F4M seems to have a stronger ability to synthesize magnetosomes than F4C under higher H_2_O_2_ concentrations. Compared with the F4C strain, the F4M strain improved resistance to oxidative stress. These observations illustrate that the sulphur-rich centre is sensitive to the oxygen concentration in MSR-1 cells. Magnetosome-forming cells must absorb large amounts of iron from the environment, and this process induces a high level of intracellular oxidative stress. Fur might function as a cellular oxygen sensor during evolution, adjusting the balance between iron and oxygen metabolism with the purpose of protecting MSR-1 cells.

Magnetosomes are difficult to create by artificial magnetic particles^42^. Due to their unique characteristics, magnetosomes have been used in many applications, such as magnetic separation of biomolecules, drug delivery, early diagnosis and detection of pathogens, hyperthermia treatment of cancer cells and magnetic resonance imaging^21^. Culturing magnetotactic bacteria in sufficient quantities to obtain large amounts of high-quality magnetosomes can be difficult, as these bacteria can only be synthesized under microaerobic conditions with sufficient iron^43^. The modified F4M strain is more resistant to oxidation. Thus, our results provide information for engineering magnetotactic bacteria by structural approaches, with far-reaching potential applications.

Fur controls the expression of toxins in pathogens and plays a vital role in host/parasite interactions^7^,^8^,^9^. As no Fur homologues are found in eukaryotes, Fur may be a potential target for the design of antimicrobial agents. In conclusion, our findings uncover the DNA recognition mechanism employed by Fur and provide the molecular basis for designing antimicrobial agents.

## Methods

### Protein expression and purification

The *fur* gene was PCR-amplified from *M. gryphiswaldense* and subcloned into a modified pET28a vector, in which the thrombin recognition site was replaced by the tobacco etch virus protease recognition site. The mutants were generated by site-directed mutagenesis. All plasmids were verified by DNA sequencing. Proteins were expressed in *E. coli* strain BL21 (DE3). Full-length and selenomethionine-containing Fur proteins were overexpressed and purified according to the reference^44^. Mutant proteins were overexpressed and purified by the same procedure. To obtain crystals of the protein–DNA complex, proteins were mixed with DNA fragments at an approximate molar ratio at 4 °C for 2 h. Complexes were purified by gel filtration chromatography (Superdex-200 10/30, GE Healthcare, UK) with a buffer of Tris-HCl (20 mM, pH 8.0), NaCl (100 mM) and MnCl_2_ (1 mM).

### DNA preparations

All DNA oligonucleotides were synthesized by Shanghai Invitrogen. DNA duplexes used for crystallization and SPR experiments were first annealed in a buffer of Tris-HCl (10 mM, pH 8.0) and NaCl (100 mM) by heating the mixture at 90 °C for 5 min and slowly cooling to room temperature over 2 h.

### Crystallization

Crystals of apo-Fur and selenomethionyl-derivative proteins were obtained using a reservoir consisting of 1.8 M (NH_4_)_2_SO_4_ and 0.1 M sodium citrate at pH 5.5, 1.4 M (NH_4_)_2_SO_4_ and 0.1 M sodium citrate at pH 5.5, respectively^44^. To obtain holo-Fur for crystallization, MnCl_2_ (final concentration: 1 mM) was added to the buffer, and the protein was concentrated to 10 mg ml^−1^. Crystals were obtained with the sitting-drop vapour-diffusion method over 10% PEG3000 in CHES (0.1 M, pH 9.0).

Different DNA fragments were attempted for crystallization of the Fur–DNA complex. Only one attempted sequence (5′-TTAATTGCAAATCATTTGCAATTGC-3′) gave crystals with an acceptable diffraction. Crystals were grown at 16 °C by the sitting-drop vapour-diffusion method. Crystals appeared in the mother solution (30% PEG4000, 0.1 M Tris-HCl pH 8.0 and 200 mM Li_2_SO_4_) and grew to full-size in 3 to 4 days. A solution of ethylene glycol (25% (v/v)) was added to the reservoir solutions as cryoprotectant, and then the crystals were frozen in liquid nitrogen until the X-ray diffraction study. Data were indexed, integrated and scaled with HKL2000 (ref. 45). Data collection and processing statistics are shown in Table 1.

### Structure determination

The apo-Fur structure was determined by selenomethionine-based single-wavelength anomalous diffraction. The initial phase was obtained by the program autoSHARP^46^. The figure of merit from the diffraction phasing was 0.38. After density modification and automatic building, six chains with 253 residues were docked with an *R*_work_ of 24.1% and *R*_free_ of 27.3%. The model was manually built using the program COOT^47^, and refinement was performed with REFMAC5 (ref. 48). The structure was refined to 1.55 Å with an *R*_work_ of 18.9% and *R*_free_ of 20.4%.

The two molecules of holo-Fur protein were located by molecular replacement with PHASER^49^, using the DBDs and DDs from apo-Fur as independent search models. Model refinement was carried out with REFMAC5 (ref. 48), followed by manual rebuilding with 2*F*_o_*−F*_c_ and *F*_o_*−F*_c_ maps. The structure was refined to 1.9 Å with an *R*_work_ of 20.3% and *R*_free_ of 22.7%.

By using the structure of holo-Fur as an input model, structures of the complex of holo-Fur with DNA were successfully solved by molecular replacement in PHASER. The position of the protein was confirmed by the anomalous signal of the SeMet-Fur-Mn^2+^–*feoAB1* operator. Model refinement was carried out with REFMAC5 (ref. 48). DNA molecules were included in the final stages of refinement. Difference Fourier maps clearly showed electron densities for bound DNA. Registry of the DNA base pairs was determined based on the shape of the electron density corresponding to the DNA bases. The final model of the Fur–*feoAB1* operator was refined to 2.6 Å with an *R*_work_ of 23.2% and *R*_free_ of 26.5%. The final model of the Fur–*P. aeruginosa* Fur box was refined to 2.75 Å with an *R*_work_ of 22.6% and *R*_free_ of 24.9%. For the structure of the Fur–*P. aeruginosa* Fur box complex, one asymmetric unit contained one Fur dimer and one single-stranded DNA molecule. The structure of one dsDNA fragment binding two Fur dimers was obtained by a twofold axis using a symmetric operation.

### Electrophoretic mobility shift assays (EMSA)

DNA (30 pmol) was incubated with protein (600 pmol) in HEPES (40 mM, pH 7.0), NaCl (125 mM) and FeSO_4_ or MnCl_2_ or EDTA (2 mM) at 25 °C for 1 h. Samples were analysed on native polyacrylamide gels (10% (v/v)) containing 0.5 × Tris-borate buffer and visualized by staining with ethidium bromide. In the EMSA competition experiment, digoxigenin (DIG)-labelled DNA (0.5 pmol) and unlabelled DNA (0, 15 or 25 pmol) were incubated with protein (1 pmol) at 25 °C for 30 min. Samples were analysed on native polyacrylamide gels (6% (v/v)) containing 0.5 × Tris-borate buffer, and were transferred to a nylon membrane for detection with the DIG Gel Shift Kit (Roche, Mannheim, Germany). The nylon membrane was exposed to an X-ray film for 15 min.

### DNase I footprinting assays

DNA fragments were prepared by PCR with a fluorescent dye FAM-labelled primers, and the PCR products were purified by agarose gel. Labelled DNA fragments (400 ng) and their respective proteins were added to a final reaction volume of 50 μl with buffer (50 mM Tris-HCl, pH 7.5, 200 mM NaCl and 5 mM MnCl_2_) and incubated at 25 °C for 1 h. DNase I (0.01 U) digestions were carried out for 1 min at 25 °C. Digestions were stopped by adding EDTA to the volume and heating the mixture at 90 °C for 10 min. DNA fragments were extracted by phenol–chloroform and precipitated by ethanol. Samples were loaded with an internal lane size standard (ROX-500, Applied Biosystems) in a 3730 DNA Genetic Analyser (Applied Biosystems). Results were analysed with GeneMarker (SoftGenetics, LLC, USA).

### Circular dichroism spectroscopy

Circular dichroism (CD) spectra of various proteins were collected on beamline VUV of the Beijing Synchrotron Radiation Facility at 16 °C. All proteins were changed to a buffer solution (10 mM MOPS, pH 7.0 and 100 mM (NH_4_)_2_SO_4_). Far-ultraviolet CD spectra (190−250 nm) of Fur proteins were scanned. A pure solvent baseline was measured with the same cell and subtracted. All spectra were processed by the CDtool software package^50^. The machine unit (mdeg) was converted into the per residue molar absorption unit, delta epsilon (Δ*ɛ*) in M cm^−1^, by normalization with respect to polypeptide concentration and path length.

### Isothermal titration calorimetry (ITC)

Experiments were performed on a Nano ITC instrument (TA Instruments) at 20 °C. WT or mutant Fur proteins were diluted (60 μM) and titrated against a buffer of Tris-HCl (20 mM, pH 8.0), NaCl (500 mM) and MnCl_2_ (2–10 mM). ITC data were processed with NanoAnalyze software.

### Differential scanning calorimetry (DSC)

DSC experiments were performed with a DSC calorimeter from MicroCal (Northampton, MA, USA) at a scan rate of 1.0 °C min^−1^. Buffer (20 mM Tris-HCl, pH 7.5 and 500 mM NaCl) was used in the reference cell of the calorimeter. Proteins were dialyzed on the buffer and used at a concentration of 1.5 mg ml^−1^. Data were analysed with the MicroCal DSC standard analysis software.

### Biosensor-binding assays

Binding of Fur proteins and DNA was detected by the SPR biosensor technique with the ProteOn XPR36 system (Bio-Rad). Biotin-labelled DNAs were captured on an NLC sensor chip (40–50 response units). Binding was performed at 25 °C in buffer containing Tris-HCl (20 mM, pH 8.0), NaCl (150 mM), MnCl_2_ (1 mM) and Tween20 (0.005% (v/v)). WT or mutant Fur proteins at different concentrations were injected over the DNA surface and blank flow cell for 3 min at a flow rate of 80 μl min^−1^. All data were collected, processed and analysed in the integrated ProteOn Manager software (Bio-Rad Laboratories) with the 1:1 Langmuir binding model.

### Construction of *fur*-mutant complement strains

*Fur*-mutant fragments were subcloned into the pRK415 vector by using the *Hin*d III and *Eco*R I sites. *E. coli* S17-1 containing the appropriate plasmid and *fur*-defective mutant strain F4 were the donor and recipient strains of biparental conjugation, respectively. The appropriate pRK415 plasmids containing *fur*-mutant fragments were introduced into F4, and the conjugants were screened by Gm^r^Tc^r^Ni^r^ colonies. Complement strains were confirmed by colony PCR.

### Quantitative real-time PCR (qPCR)

WT and *fur*-mutant strains were grown in sodium lactate medium (SLM) to an optical density (OD)_565_ of 0.7. The culture was split, with half being added to 2,2′-dipyridyl (30 μM DIPy; low-iron condition) and half to ferric citrate (60 μM; high-iron condition). Growth was continued for 2 h at 30 °C, and the cells were harvested. Total cellular RNA was isolated with Trizol reagent (TIANGEN Biotech Co., Ltd, Beijing, China) and digested with RNase-free DNase I (TaKaRa, Japan) for 2 h at 37 °C. The ratio of A_260_/A_280_ was used to evaluate RNA quality and quantity using the NanoVue Spectrophotometer (GE Healthcare).

RNA extracted from each sample was reverse-transcribed into complementary DNA with M-MLV reverse transcriptase (Promega, USA). RNA (2 μg) was mixed with random primer (2 μl), and double-distilled H_2_O (ddH_2_O) was added to reach the final volume (12 μl). The sample was incubated at 70 °C for 10 min, followed by 2 min on ice immediately thereafter. Then, M-MLV reverse transcriptase (1 μl), M-MLV RT 5 × buffer (4 μl), inhibitor (0.5 μl, TaKaRa), dNTP (1.25 μl of 10 mmol) and ddH_2_O (1.25 μl) were added to the 12-μl mixture. The reaction occurred sequentially at 30 °C for 10 min, 42 °C for 1 h and 70 °C for 15 min.

Quantitative PCR was performed in a LightCycler 480 RT-PCR System (Roche, USA), using the LightCycler 480 SYBR Green I Master Kit according to the manufacturer's instructions (Roche). Specific primers (Supplementary Table 3) were designed to yield 150- to 250-bp products. A PCR system (20 μl) was prepared, which contained complementary DNA templates (2 μl) from different samples, each primer (2 μl of 5 μM), SYBR Green I Mix (10 μl) and water (4 μl). The PCR procedure was performed according to the manufacturer's instruction. For a negative control, template DNA was replaced by PCR-grade water. Specificity of the amplified PCR product was assessed by performing a melting curve analysis on the LightCycler 480 Instrument. The *rpoC* gene encoding RNA polymerase subunit β′ was chosen as the internal control and reference^51^.

To analyse the results, the relative expression of each gene was calculated by the comparative crossing point (Cp) method. Relative expression was presented by the formula 2^–ΔΔCp^. The results displayed the expression fold changes between MSR-1 cells treated with the iron chelator DIPy (30 μM) and the cells treated with ferric citrate (60 μM). Data from three replicates were averaged.

### Cell growth and TEM

WT, F4 (*fur* mutant), F4C (*fur* complement stain) and other *fur*-mutant complement strains (Supplementary Table 4) were cultured in SLM with ferric citrate (20 μM) at 30 °C, 100 r.p.m. for 24 h. OD_565_ was measured by a ultraviolet–vis spectrophotometer (UNICO2100, UNICO Instrument Co., Ltd, Shanghai, China). Cmag was calculated by measuring the maximum and minimum scattering intensities^52^. Cells of each strain were coated on copper or carbon grids and washed twice with ddH_2_O. Samples were observed directly by TEM (JEM-1230, JEOL, Japan).

### Tolerance to H_2_O_2_, SNG and iron content of strains

Strains (WT, F4, F4C and F4M) were grown in SLM (100 ml) supplemented with H_2_O_2_ (200, 500 or 800 μM) at 30 °C. After 24 h, OD_565_ and Cmag were measured. SNG was prepared as a stock solution (1 mg ml^−1^) in dimethyl sulfoxide (DMSO). Strains (WT, F4, F4C and F4M) were cultured in SLM at 30 °C until the stationary phase. Cultures with added SNG (1 μg ml^−1^) or with the equivalent concentration of DMSO as a control were incubated in a rotary shaker (100 r.p.m., 3 h, 30 °C) and serially diluted 10-fold. An aliquot (10 μl) of each dilution was spotted on an agar plate with sodium lactate-sodium glutamate medium and incubated 7 days at 30 °C. Strains (WT, F4, F4C and F4M) were cultured in SLM (100 ml) supplemented with ferric citrate (20, 40 or 60 μM) at 30 °C for 24 h. The total intracellular iron content was measured by ICP-OES (Optima 5300DV; Perkin-Elmer, Waltham, MA, USA)^53^.

### DNA shape analysis

Bound DNA conformations in the crystal structures were analysed with CURVES 5.3 (ref. 54) where the values for all levels assigned to the same nucleotide were averaged^24^. Hydroxyl radical-cleavage intensities representing an experimental measure for minor groove width in unbound DNA were derived with ORChID2 (ref. 27), as implemented in GBshape^55^.

### Electrostatic potential calculation

Electrostatic potential was calculated for DNA at a physiologic ionic strength of *I*=0.145 M in the absence of protein by using the Poisson–Boltzmann solver DelPhi^54^, based on a previously described protocol^24^. To express the electrostatic potential as a function of sequence, reference points in the centre of the minor groove in approximately the plane of base pair *i* were defined as geometric midpoints between the O4′ atoms of nucleotides *i+1* in strand I and nucleotide *i−1* in strand II^24^.

## Additional information

**Accession codes:** The Protein Data Bank (PDB) accession numbers in this paper are 4RAY, 4RAZ, 4RB0, 4RB1, 4RB2 and 4RB3.

**How to cite this article:** Deng, Z. *et al.* Mechanistic insights into metal ion activation and operator recognition by the ferric uptake regulator. *Nat. Commun.* 6:7642 doi: 10.1038/ncomms8642 (2015).

## Supplementary Material

Supplementary Figures and Tables Supplementary Figures 1-7, Supplementary Tables 1-4, Supplementary references

Supplementary Movie 1 Mechanism of Fur regulation by transition metal ions and DNA.

## Figures and Tables

**Table 1:** Data collection and refinement statistics of MSR-1 Fur and complexes.

	**SeMet-apo-Fur**	**Apo*****-*****Fur**	**Holo-Fur**	**SeMet-Fur-Mn**^2+^**–*****feoAB1*** **operator**	**Fur-Mn**^2+^**–*****feoAB1*** **operator**	**Fur-Mn**^2+^**–*****P. aeruginosa*** **Fur box**
*Data collection*						
Space group	*C*2	*C*2	*I*222	*C*2	*C*2	*C*2
Cell dimensions						
*a*, *b*, *c* (Å)	69.2, 78.5, 66.1	69.4, 78.2, 66.5	79.6, 96.8, 119.9	94.3, 68.7, 82.6	94.0, 68.6, 84.0	159.5, 42.9, 60.5
*α*, *β*, *γ* (˚)	90, 108.9, 90	90, 108.7, 90	90, 90, 90	90, 109.3, 90	90, 108.7, 90	90, 94.9, 90
Resolution (Å)*	50–1.85 (1.88–1.85)	50–1.55 (1.58–1.55)	50–1.90 (1.93–1.9)	50–2.82 (2.87–2.82)	50–2.6 (2.64–2.6)	50–2.75 (2.8–2.75)
*R*_merge_ (%)	5.4 (23.8)	5.7 (58.3)	6.9 (65.3)	7.8 (77.9)	8.2 (59.5)	8.3 (64.7)
*I*/σ*I*	52.5 (6.1)	44.4 (2.9)	44.4 (2.8)	26.9 (2.2)	43.6 (2.2)	22.5 (2.1)
Completeness (%)	98.1 (90.0)	99.5(100.0)	99.5 (95.2)	91.4 (92.4)	97.1 (85.4)	94.5 (96.5)
Redundancy	7.3 (5.7)	5.1 (5.0)	9.3 (5.4)	6.2 (6.1)	10.4 (6.2)	8.8 (7.9)
						
*Refinement*						
Resolution (Å)	50–1.85 (1.90–1.85)	50–1.55 (1.59–1.55)	50–1.9 (1.95–1.9)	45.73–2.82 (2.89–2.82)	44.56–2.6 (2.67–2.6)	30–2.75 (2.82–2.75)
No. of reflections	26,458 (1,572)	45,803 (3,295)	34,732 (2,277)	10,544 (772)	14,488 (920)	8,112 (432)
*R*_work_/*R*_free_ (%)	17.4/20.7 (16.3/28.8)	18.9/20.4 (22.5/26.3)	20.3/22.7 (37.0/39.7)	23.8/27.5 (39.6/35.3)	23.2/26.5 (41.3/49.9)	22.6/24.9 (34.2/36.4)
No. of atoms						
Protein	2,094	2,134	2,216	1,998	2,102	1,985
DNA/ion	72	33	16	1,059	1,028	487
Water	97	135	190	14	15	3
*B*-factors						
Protein	31.0	28.3	34.3	89.6	77.0	45
DNA/ion	31.6	31.5	34.7	91.2	75.6	34.2
Water	32.2	34.2	40.3	72.8	61.2	29
r.m.s.d.						
Bond lengths (Å)	0.008	0.006	0.010	0.004	0.005	0.007
Bond angles (°)	1.467	1.400	1.293	0.866	0.944	1.340
Ramachandran plot (%)†	95.2/4.3/0.4/0.1	95.5/4.5/0/0	93.8/6.2/0/0	89.2/10.8/0/0	88.1/11.9/0/0	88.6/11.4/0/0

r.m.s.d., root mean squared deviation.

Note that all Fur protein in complex structures is C9L/M14L/M16V-triple mutant with the exception of SeMet-Fur-Mn^2+^–*feoAB1* operator.

^*^Statistics for the highest-resolution shell.

^†^Residues in most favoured, additional allowed, generously allowed and disallowed regions of the Ramachandran plot.
