# Supplementary material for: Mechanistic insights into metal ion activation and operator recognition by the ferric uptake regulator
Source: Nat Commun. 2015 Jul 2;6:7642. doi: 10.1038/ncomms8642 (PMC4506495; doi:10.1038/ncomms8642)
Supplement: Supplementary Figures and Tables — Supplementary Figures 1-7, Supplementary Tables 1-4, Supplementary references [file ncomms8642-s1.pdf]

**a**

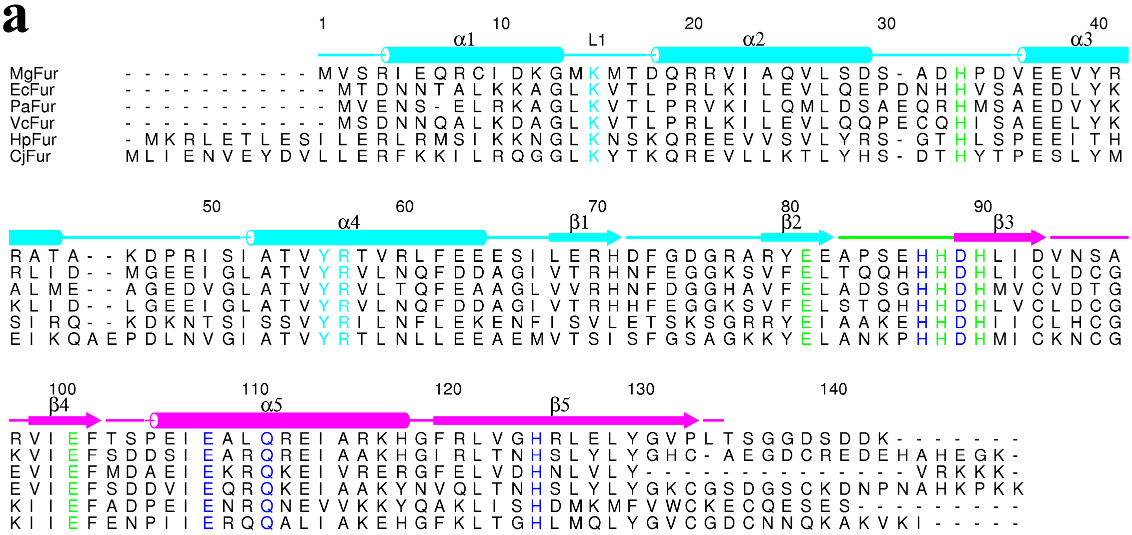
1 10 20 30 40
α1 L1 α2 α3
MgFur -----------MVSRIEQRCIDKGMKMTDQRRVIAQVLSDS-ADHPDVEEVYR
EcFur ------------MTDNNTALKKAGLKVTLPRLKILEVLQEPDNHHVSAEDLYK
PaFur ------------MVENS-ELRKAGLKVTLPRVKILQMLDSAEQRHMSAEDVYK
VcFur ------------MSDNNQALKDAGLKVTLPRLKILEVLQQPECQHISAEELYK
HpFur -MKRLETLESILERLRMSIKKNGLKNSKQREEVVSVLYRS-GTHLSPEEITH
CjFur MLIENVEYDVLLERFKKILRQGGLKYTKQREVLLKTLYHS-DTHYTPESLYM
50 60 70 80 90
α4 β1 β2 β3
RATA--KDPRISIATVYRTVRLFEEESILERHDFGDGRARYEEAPSEHHDHLIDVNSA
RLID--MGEEIGLATVYRVLNQFDDAGIVTRHNFEGGKSVFELTQQHHHDHLICLDCG
ALME--AGEDVGLATVYRVLTQFEAAGLVVRHNFDGGHAVFELADSGHHDHMVCVDTG
KLID--LGEEIGLATVYRVLNQFDDAGIVTRHHFEGGKSVFELSTQHHHDHLVCLDCG
SIRQ--KDKNTSISSVYRILNFLEKENFISVLETSKSGRRYEIAAKEHHDHIICLHCG
EIKQAEPDLNVGIATVYRTLNLLEEAEMVTSISFGSAGKKYELANKPHHDHMICKNCG
100 110 120 130 140
β4 α5 β5
RVIEFTSPEIEALQREIARKHGFRLVGHRLELYGVPLTSGGDSDDK-------
KVIEFSDDSIEARQREIAAKHGIRLTNHSLYLYGHC-AEGDCREDEHAHEGK-
EVIEFMDAEIEKRQKEIVRERGFELVDHNLVLY------------VRKKK-
EVIEFSDDVIEQRQKEIAAKYNVQLTNHSLYLYGKCGSDGSCKDNPNAHKPKK
KIIEFADPEIENRQNEVVKKYQAKLISHDMKMFVWCKECQESES---------
KIIEFENPIIERQQALIAKEHGFKLTGHLMQLYGVCGDCNNQKAKVKI-----

**b**

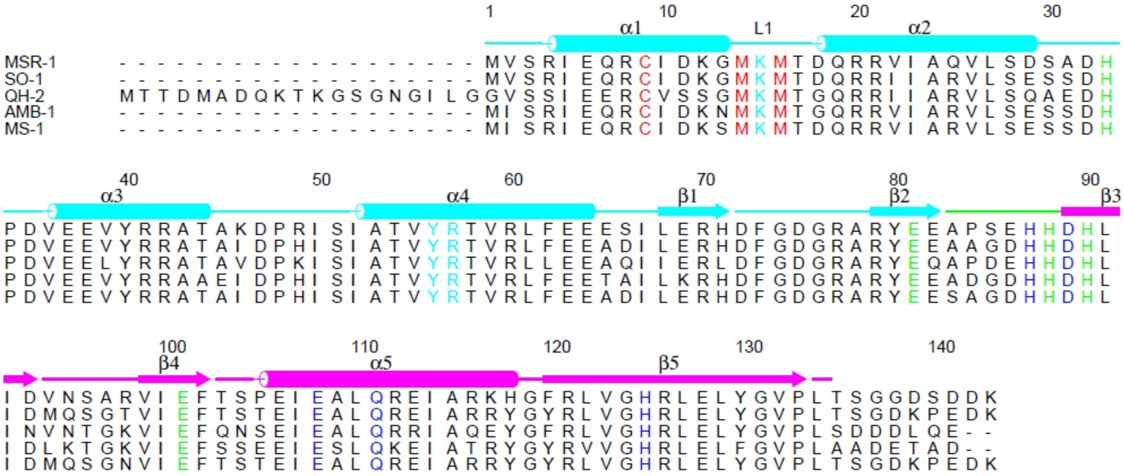
1 10 20 30
α1 L1 α2
MSR-1 --------------------MVSRIEQRCIDKGMKMTDQRRVIAQVLSDSADH
SO-1 --------------------MVSRIEQRCIDKGMKMTDQRRIIARVLSESSDH
QH-2 MTTDMADQKTKGSGNGILGGVSSIEERCVSSGMKMTGQRRIIARVLSQAEDH
AMB-1 --------------------MISRIEQRCIDKNMKMTGQRRVIARVLSESSDH
MS-1 --------------------MISRIEQRCIDKSMKMTDQRRVIARVLSESSDH
40 50 60 70 80 90
α3 α4 β1 β2 β3
PDVEEVYRRATAKDPRISIATVYRTVRLFEEESILERHDFGDGRARYEEAPSEHHDHL
PDVEEVYRRATAIDPHISIATVYRTVRLFEEADILERHDFGDGRARYEEAAGDHHDHL
PDVEELYRRATAVDPKISIATVYRTVRLLEEAQIIERLDFGDGRARYEQAPDEHHDHL
PDVEEVYRRAAEIDPHISIATVYRTVRLFEETAILKRHDFGDGRARYEEADGDHHDHL
PDVEEVYRRATAIDPHISIATVYRTVRLFEEADILERHDFGDGRARYEESAGDHHDHL
100 110 120 130 140
β4 α5 β5
IDVNSARVIEFTSPEIEALQREIARKHGFRLVGHRLELYGVPLTSGGDSDDK
IDMQSGTVIEFTSTEIEALQREIARRYGYRLVGHRLELYGVPLTSGDKPEDK
INVNTGKVIEFQNSEIEALQRRIAQEYGFRLVGHRLELYGVPLSDDDLQE--
IDLKTGKVIEFSSEEIESLQKEIATRYGYRVVGHRLELFGVPLAADETAD--
IDMQSGNVIEFTSTEIEALQREIARRYGYRLVGHRLELYGVPLTSGDKPEDK

**c**

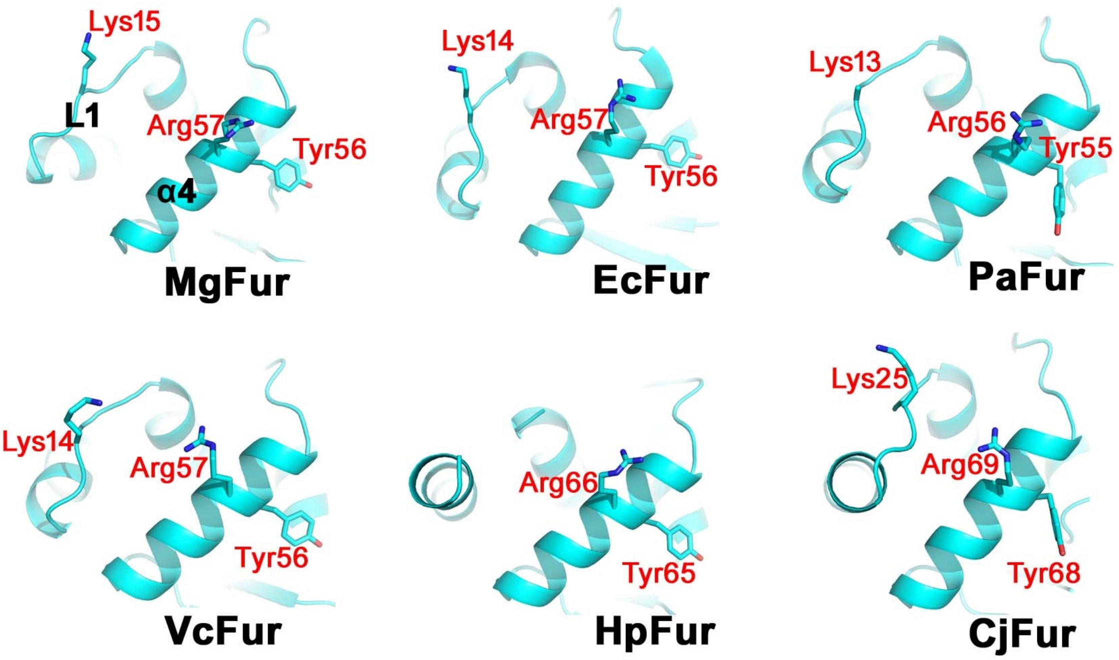


**Supplementary Figure 1. Sequence alignment of prokaryotic Fur proteins.** (**a**) The secondary structure elements of MgFur are shown above the alignment and colored by domains as in Figure 2a. The N-terminal DBD, hinge and C-terminal DD are shown in cyan, green and magenta, respectively. Residues of binding site 1 and binding site 2 are colored in green and blue, respectively. Residues that are important for interacting with the DNA are colored in cyan. (**b**) Sequence alignment of Fur proteins from five high homology *Magnetospirillum* or *Magnetospira* strains. The sequences used are as follows: *M. gryphiswaldense* MSR-1, *Magnetospirillum* sp. SO-1 (AONQ00000000), *Magnetospira* sp. QH-2 (FO538765), *M. magneticum* AMB-1 (AP007255), *M. magnetotacticum* MS-1 (AAAP00000000). The red colored letters show the conserved sulfur-containing amino acids in *Magnetospirillum* and *Magnetospira* strains. (**c**) Superimposition of the DBDs of different prokaryotic Fur proteins. The DBDs are shown in the same orientation. Note that the lysine residue was not shown in structures of HpFur due to its flexible conformations in the holo-Fur structures. Sequences and structures used: *M. gryphiswaldense* (Mg, this study), *E. coli* (Ec; PDB ID: 2FU4), *P. aeruginosa* (Pa; PDB ID: 1MZB), *V. cholerae* (Vc; PDB ID: 2W57), *H. pylori* (Hp; PDB ID: 2XIG), *C. jejuni* (Cj; PDB ID: 4ETS).

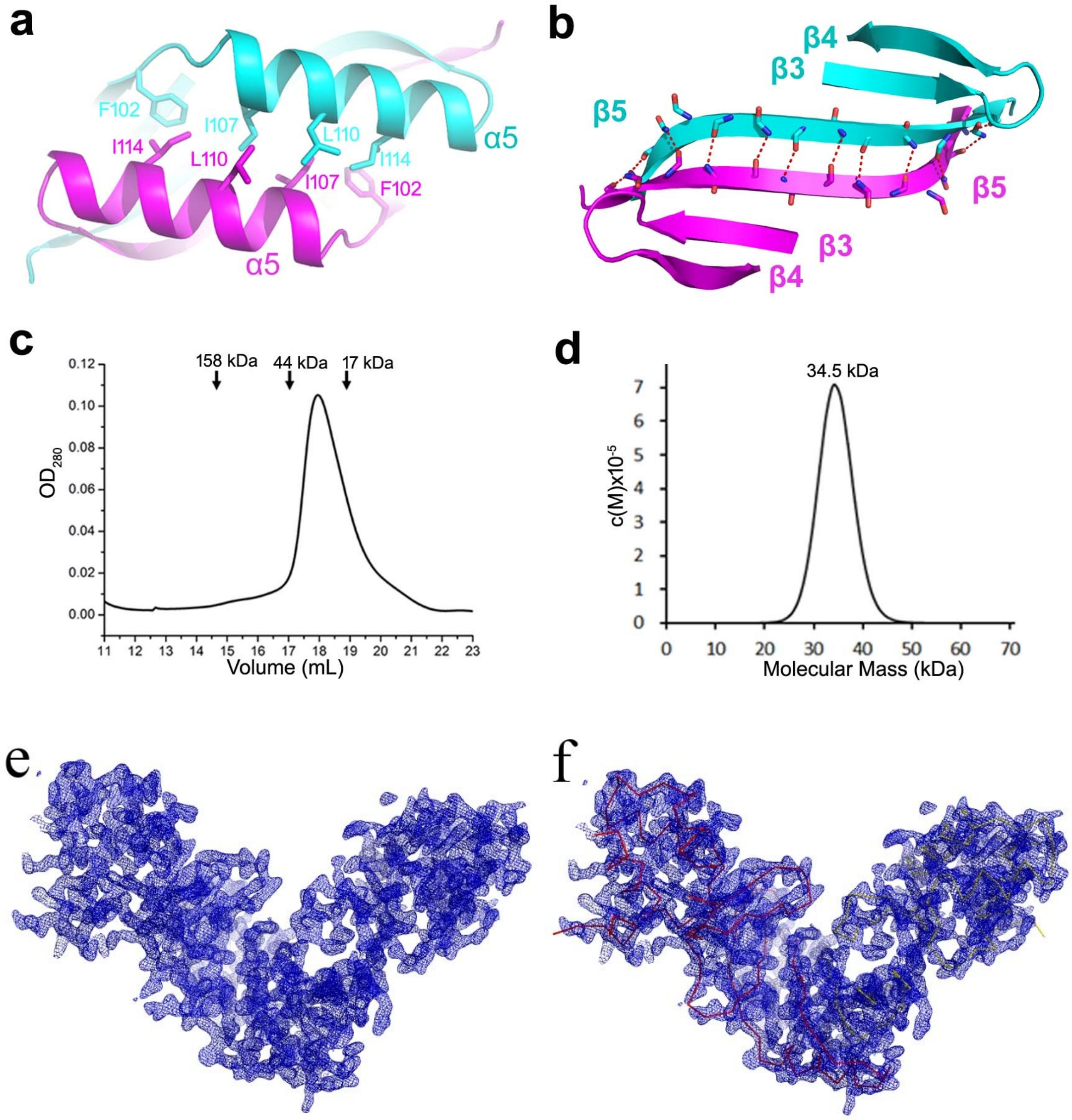


**Supplementary Figure 2. Apo-Fur forms a dimer in crystal and solution.** (**a**) Hydrophobic interactions between the two antiparallel helices α5 in the two monomers. (**b**) Two antiparallel β5 strands form hydrogen bonds. (**c**) Elution profile of apo-Fur by size-exclusion chromatography. The elution volumes of the molecular mass standards are marked at the top of the panel. (**d**) Analytical ultracentrifugation of apo-Fur, c(s) distribution from sedimentation velocity analytical ultracentrifugation experiments (SV) performed at 0.1 mM protein. Note that the theoretical mass of Fur monomer is 16.4 kDa. (**e**) Experimental electron density map of holo-Fur. (**f**) Experimental electron density map of holo-Fur with solved backbone traces of holo-Fur. The two molecules of holo-Fur in one asymmetric unit in the *I*222 space group. The electron density is contoured at 2.0 σ. The backbone traces of the two molecules are colored in different colors.

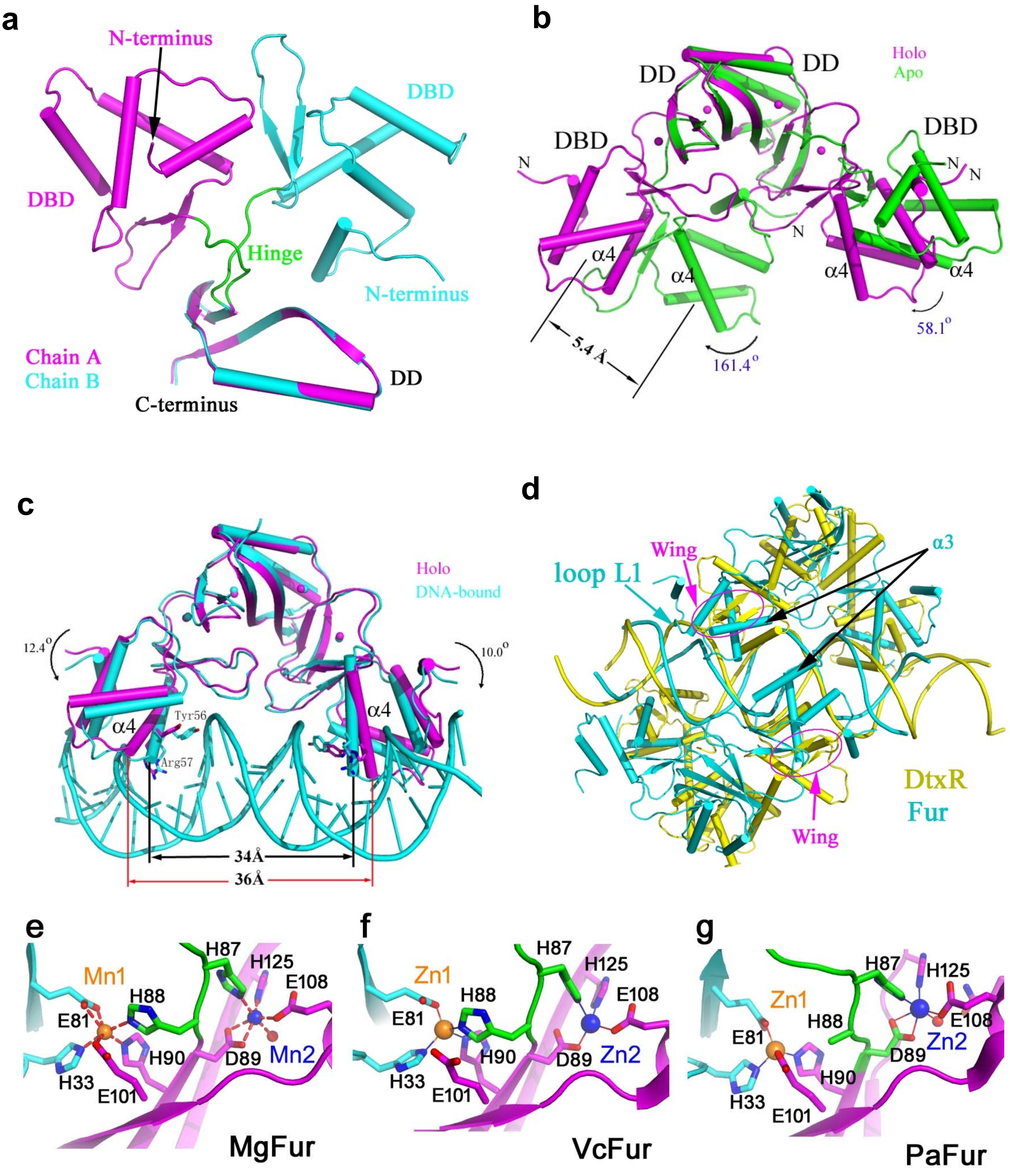


**Supplementary Figure 3. Comparison of different states of Fur**. (**a**) Comparison of the two monomer conformations in apo-Fur. (**b**) Side view of the recognition helix α4 to highlight the conformational change of the DBD. The key residues Tyr56 and Arg57 are shown as sticks and the distances between the two helices α4 in holo-Fur (magenta) and the DNA-bound form (cyan) are labeled. The arrow indicates the movement of the DBD in the DNA-bound state compared to the holo state. In addition, two DBDs decrease their distance from each other after binding to the *feoAB1*

operator. (**c**) Comparison of holo-Fur and the Fur-*feoAB1* operator complex. (**d**) Comparison of the DtxR-*tox* operator (PDB ID: 1DDN) and Fur-*P. aeruginosa* Fur box complex. Note that the wing of DtxR is shown by a magenta arrow. The wing of DtxR has an anti-parallel β-sheet, which is not found in the corresponding position of Fur. Coordination of the two metal ion binding sites in (**e**) MgFur, (**f**) VcFur (PDB ID: 2w57), and (**g**) PaFur (PDB ID: 1MZB). To make the comparison clear, the residue number is not changed for PaFur (The actual residue number of PaFur is the labeled number minus one).

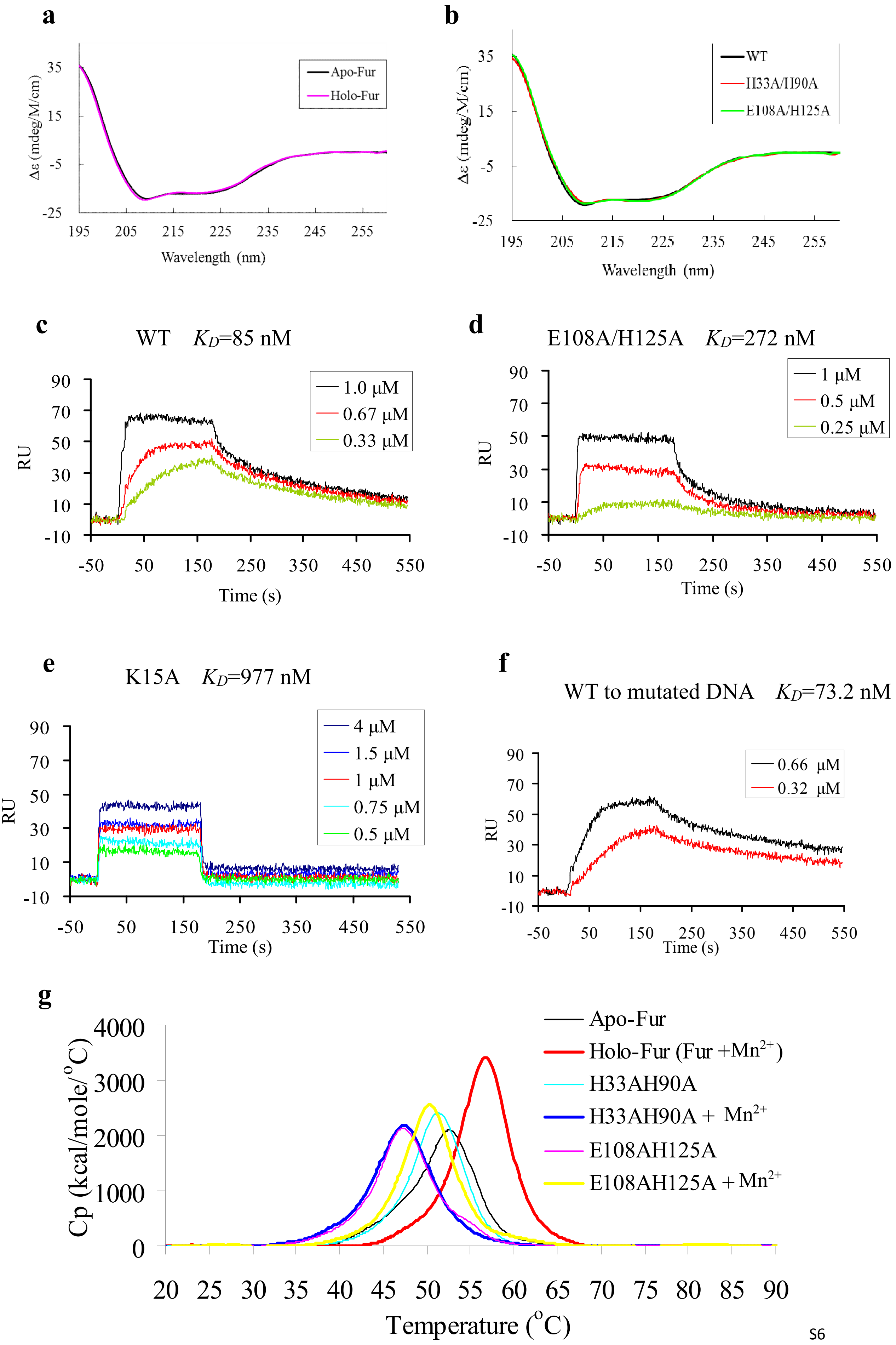

a
35
15
-5
-25
Δε (mdeg/M/cm)
195 205 215 225 235 245 255
Wavelength (nm)
Apo-Fur
Holo-Fur
b
35
15
-5
-25
Δε (mdeg/M/cm)
195 205 215 225 235 245 255
Wavelength (nm)
WT
H33A/H90A
E108A/H125A
c
WT KD=85 nM
90 70 50 30 10 -10
RU
-50 50 150 250 350 450 550
Time (s)
1.0 μM
0.67 μM
0.33 μM
d
E108A/H125A KD=272 nM
90 70 50 30 10 -10
RU
-50 50 150 250 350 450 550
Time (s)
1 μM
0.5 μM
0.25 μM
e
K15A KD=977 nM
90 70 50 30 10 -10
RU
-50 50 150 250 350 450 550
Time (s)
4 μM
1.5 μM
1 μM
0.75 μM
0.5 μM
f
WT to mutated DNA KD=73.2 nM
90 70 50 30 10 -10
RU
-50 50 150 250 350 450 550
Time (s)
0.66 μM
0.32 μM
g
4000 3000 2000 1000 0
Cp (kcal/mole/°C)
20 25 30 35 40 45 50 55 60 65 70 75 80 85 90
Temperature (°C)
Apo-Fur
Holo-Fur (Fur +Mn2+)
H33AH90A
H33AH90A + Mn2+
E108AH125A
E108AH125A + Mn2+

**h**

| | $t_{1/2}$ (°C) | Δ*H* (kcal/mol) | $T_{onset}$ (°C) | $T_m$ (°C) |
|---|---|---|---|---|
| Apo-Fur | 7.38 | 18600 | 39.42 | 52.49 |
| Holo-Fur (Apo-Fur + $Mn^{2+}$) | 6.52 | 26900 | 44.46 | 56.68 |
| H33AH90A | 6.86 | 18800 | 39.2 | 51.26 |
| H33AH90A + $Mn^{2+}$ | 7.54 | 20000 | 33.03 | 47.28 |
| E108AH125A | 7.2 | 19200 | 33.93 | 47.33 |
| E108AH125A + $Mn^{2+}$ | 6.69 | 20800 | 37.61 | 50.35 |

**Supplementary Figure 4. Properties of Fur and its mutants**. (**a**) CD spectra of apo-Fur and holo-Fur in the presence of 1 mM $Mn^{2+}$. It shows that the addition of metal ions does not alter the secondary structures. (**b**) CD spectra of the WT, H33A/H90A and E108A/H125A mutants. These data demonstrate that the secondary structures of the two mutants in the metal ion binding sites are similar to that of the WT. (**c-f**) SPR experiment of Fur–*feoAB1* operator binding. Binding of WT Fur and mutants to the *feoAB1* operator, determined by surface plasmon resonance (SPR) analysis. The *FeoAB1* operator was immobilized onto an NLC sensor chip and subsequently the buffer containing each Fur at the corresponding concentrations was run over the immobilized *feoAB1* operator. $K_D$ values calculated from SPR data are also presented. (**g**) DSC profile of Cp vs. T and thermodynamic binding parameters of (**h**) Fur mutants with or without $Mn^{2+}$ obtained by subtracting the baseline from the original scan. 1.5 mg/ml protein was used for each sample. All experiments were repeated three times.

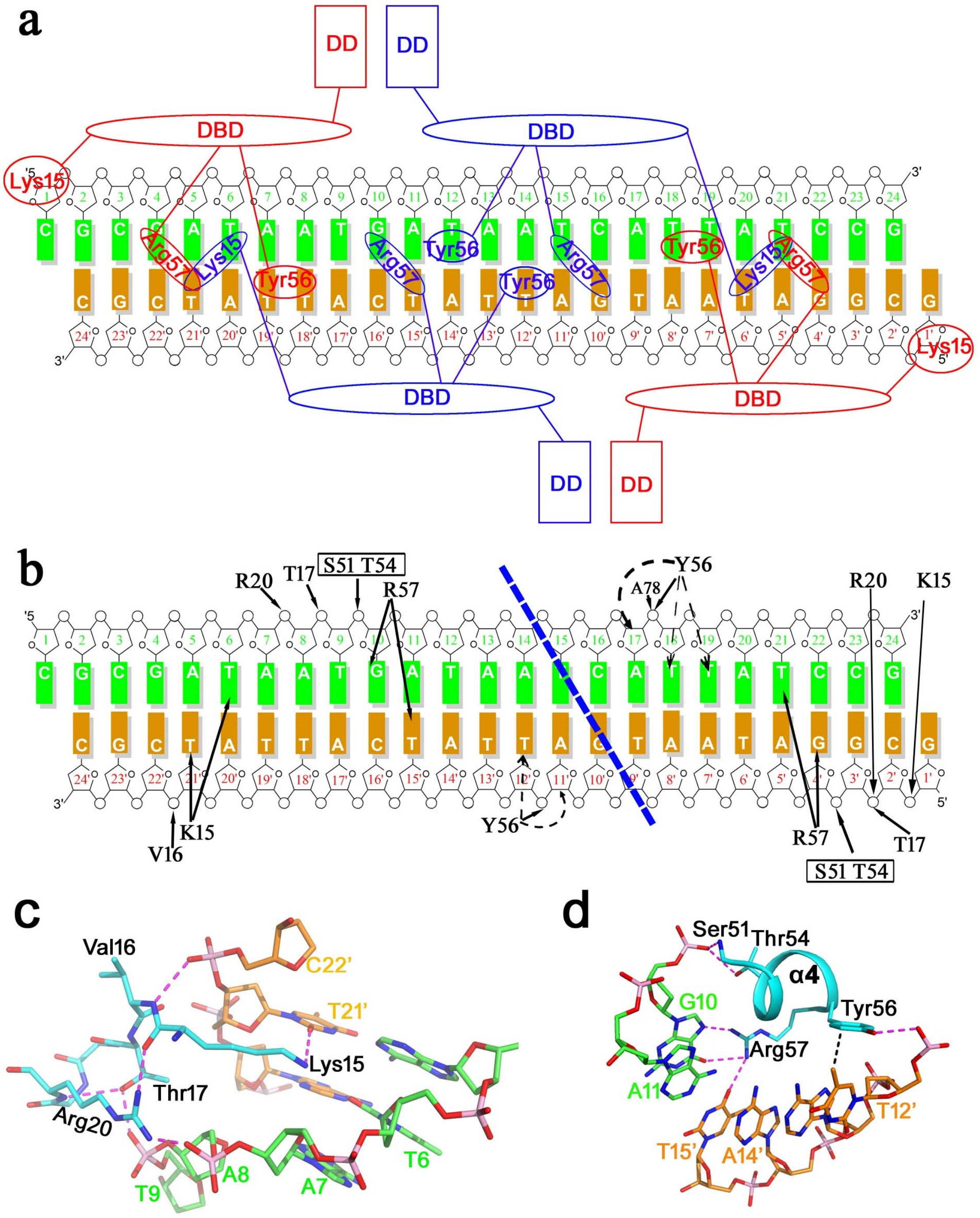
a
DD
DD
DBD
DBD
Lys15
Arg57
Lys15
Tyr56
Arg57
Tyr56
Tyr56
Arg57
Tyr56
Lys15
Arg57
Lys15
'5
3'
C G C G A T A A T G A T A A T C A T T A T C C G
C G C T A T T A C T A T T A G T A A T A G G C G
3'
5'
DBD
DBD
DD
DD
b
R20
T17
S51 T54
R57
Y56
A78
R20
K15
V16
K15
Y56
R57
T17
S51 T54
c
Val16
C22'
T21'
Lys15
Thr17
Arg20
T9
A8
A7
T6
d
Ser51
Thr54
α4
G10
Tyr56
Arg57
A11
T12'
T15'
A14'

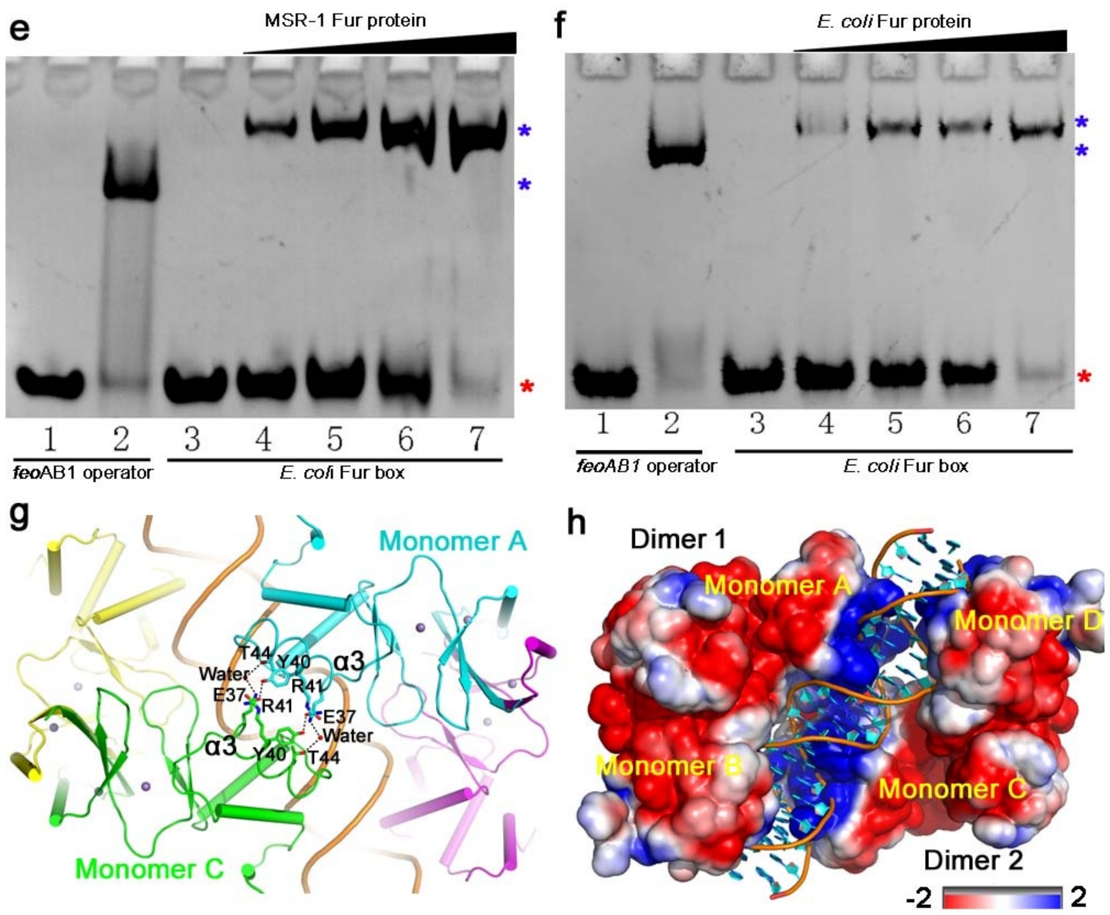


**Supplementary Figure 5. Interactions between two MgFur dimers and the *P. aeruginosa* Fur box.** (**a**) Schematic diagram of two Fur-DNA interactions. (**b**) Detailed Fur-DNA interactions between one Fur dimer with DNA. Solid arrows and dashed arrows indicate hydrogen bonds and van der Waals interactions, respectively. Another Fur dimer-DNA interaction is the same because the whole structure is related by a twofold symmetry axis. Close-up view of the interactions of loop 1 (L1) with (**c**) the minor groove and contacts between helix α4 and (**d**) the major groove. Magenta dashed lines represent hydrogen bonds and black dashed lines indicate van der Waals interactions. Note that one asymmetric unit contained one Fur dimer and one ssDNA molecule. The structure of one double stranded DNA fragment in complex with two Fur dimers was obtained by a symmetry operation using the twofold axis. (**e**) MSR-1 Fur protein and (**f**) *E. coli* Fur protein. Lane 1 and 3 are the control *feoAB1* operator or *P. aeruginosa* Fur box (25 pmol). The Fur protein concentration used in lane 2, lanes 4-7 are 100, 10, 20, 40 and 80 pmol, respectively. The corresponding protein complexes are shown as blue asterisks. Note that the *E. coli* Fur protein contained a His Tag and a TEV site. (**g**) Detailed interactions between two Fur dimers in Fur-$Mn^{2+}$-*P. aeruginosa* Fur box complex. (**h**) Electrostatic surface potential of Fur-$Mn^{2+}$-*P. aeruginosa* Fur box ternary complex using the APBS tool of PyMOL (unit: kT/e). The view is that of panel (**g**) rotated around the y-axis by 180º. The results show that two Fur dimers cooperatively bind to *P. aeruginosa* Fur box independent of protein concentration.

a

b

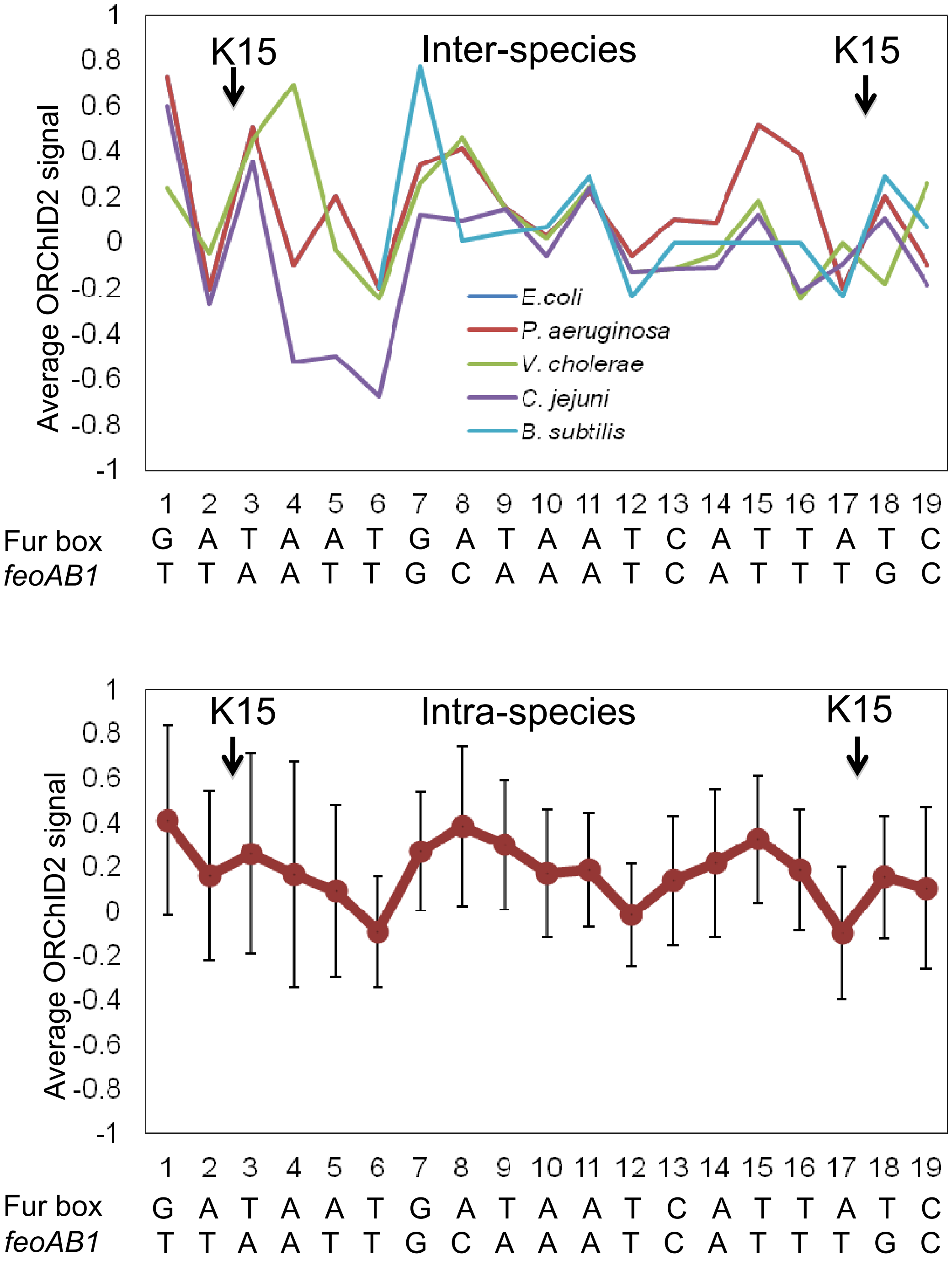


c

| Gene | Fur-box element |
|---|---|
| Consensus | GATAATGATAATCATTATC |
| pig2 | ACGAACGTGAATCATTCTC |
| pig3_1 | GATAATGAGATTGATTATT |
| pig3_2 | GAAAACAATAATCAATCTC |
| pig4_1 | GACATTGAGATTCAATGAC |

| | |
|---|---|
| pig4_2 | ATAATTGAGAATCGTTATT |
| pig6 | GAAGATGGTAATTAATTGC |
| pig7_1 | TCCAATGCAAATCAATATC |
| pig7_2 | GCCAATGATATTGATTTGC |
| pig8 | GTAATTGACAATCATTATC |
| pig9_1 | GATAATGAGAATAGTTATT |
| pig9_2 | TGTAATAATAACTATTCTC |
| pig12 | TTTATCGCAACTGATTATC |
| pig13_1 | CTGAATGATAATAATTATC |
| pig13_2 | TCTTCTGATAATTATTATC |
| pig14 | GCAAACGATATTCATCATC |
| pig17 | AAAAACGAGAATTATTCGC |
| pig18 | GATATTAATACCCATTTCA |
| pig19 | ACGATTGCTAATCAATCTT |
| pig20 | GGAAATGAGAATCATTATT |
| pig23 | CTGAATGAAACCCAATCTT |
| pig25_1 | ACGATTGAAAATCATTATC |
| pig25_2 | TTTTATGATAATGATTTTC |
| pig31 | GTTATTGAGAATCATTGGC |
| pig32_1 | CTTGATGAGAATTATTATG |
| pig32_2 | TAAATTCATAATAATTCTC |
| fpvA_1 | GCTGATCACGATGATGGTC |
| fpvA_2 | GATCACGATGATGGTCTTG |
| pfeA | TCAAATAACAATCAATATC |
| fptA | CATAATGATAAGCATTATC |
| pchR_1 | GGAAATGAGATTTATTATC |
| pchR_2 | GAGATTTATTATCATTGGC |

**Supplementary Figure 6. Hydroxyl radical cleavage intensities of unbound Fur binding sites derived with ORChID2.** (**a**) Consensus Fur-boxes among different species[1-4] and (**b**) average of 32 sequences bound by Fur from *P. aeruginosa*[1]. The different sequences were aligned to the Fur box based on the conserved nucleotides. Positions where Lys15 would interact with the DNA minor groove, based on the co-crystal structures from this study, are highlighted by an arrow. The sequences used to generate panel (**a**) are listed in Supplementary Table 1. (**c**) The Fur-box elements of iron-regulated genes in *P. aeruginosa* were used to generate panel (**b**). See Methods for ORChID2 references.

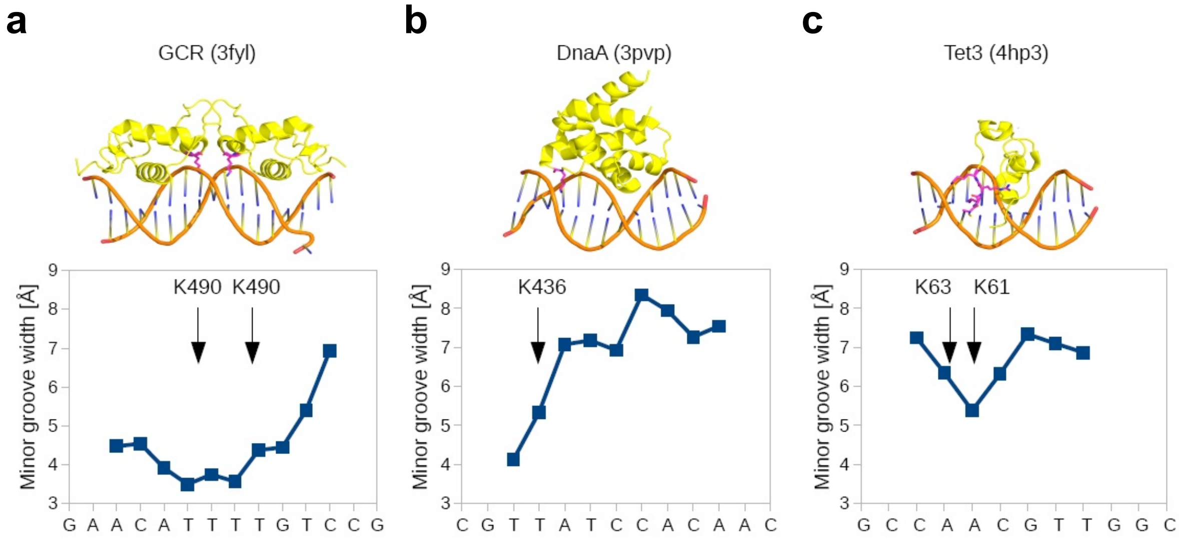


**Supplementary Figure 7. Lysine is employed to recognize minor groove shape and electrostatic potential**

The Fur-DNA ternary complexes presented in this study reveal that lysine can play a key role in DNA shape readout (Figure 6). This finding expands our currently established knowledge that predominantly arginine[5-9] and histidine[10] employ this form of protein-DNA readout. Lysine, albeit its abundance at protein surfaces, is in relative terms less frequently found to insert into narrow minor groove regions. A reason for this observation is the smaller volume of its charged side chain, resulting in a smaller Born radius of its head group compared to the ones of arginine and histidine. This results in a higher desolvation cost if its hydration shell was to be removed in order for the lysine side chain to be inserted into a narrow region of the minor groove[5].

After revealing the use of Lys15 side chains in Fur-DNA recognition, we surveyed the Protein Data Bank, which resulted, compared with arginine, in fewer examples where lysine alone has been observed to intrude a narrow minor groove, such as (**a**) the *Rattus norvegicus* glucocorticoid receptor DBD homodimer (PDB ID: 3FYL)[11], (**b**) the *Mycobacterium tuberculosis* DnaA-DBD (PDB ID: 3PVP)[12], and (**c**) the *Xenopus tropicalis* Tet3 CXXC domain (PDB ID: 4HP3)[13]. Among these examples, DnaA binds to origins of replication through the insertion of Lys436 into the minor groove, and when mutated to alanine its binding affinity decreases[12]. The same study shows that in other bacteria, however, the respective position is occupied by an arginine instead of lysine, which carries the same positive charge while its desolvation cost is lower than that of lysine[5].

Taken together, the ternary Fur-DNA complexes presented in this study revealed the use of lysine in recognizing intrinsically narrow regions of the minor groove with enhanced negative electrostatic potential, and the survey of the Protein Data Bank suggests that lysine can replace arginine in certain systems, thus expanding the currently known repertoire of DNA shape readout mechanisms.

**Supplementary Table 1. DNA consensus boxes bound by Fur from different species.** Conserved nucleotides are shown using colored font**.**

| **Species**[14] | **Sequences** |
|---|---|
| *E.coli*[15] | GATAATGATAATCATTATC |
| *P. aeruginosa*[1] | GATAATGATAATCATTATC |
| *V. cholerae*[2] | GCCAAATGATAATTATTCTCATTGC |
| *C. jejuni*[3] | TATTTTGATAATTATTATCA |
| *B. subtilis*[4] | TGATAATNATTATCA |

**Supplementary Table 2. Metal ion–ligand distances in metal ion binding sites of holo-Fur.**

| Atom | Distance (Å) | Atom | Distance (Å) |
|---|---|---|---|
| **Site 1** | | **Site 2** | |
| His-33 NE2 | 2.24 | His-87 NE2 | 2.16 |
| Glu-81 OE1 | 2.30 | Asp-89 OD1 | 2.28 |
| Glu-81 OE2 | 2.20 | Asp-89 OD2 | 2.36 |
| His-88 NE2 | 2.21 | Glu-108 OE1 | 2.05 |
| His-90 NE2 | 2.18 | His-125 NE2 | 2.24 |
| Glu-101 OE1 | 2.17 | Water | 2.26 |

**Supplementary Table 3. Primers of quantitative real-time RT-PCR.**

| Gene name | Location | Primer name | Sequence | Product length (bp) |
|---|---|---|---|---|
| *feoB1* | EF120624.1 | FeoB1-F | AAGGCGATGACGAGGTTCT | 238 |
| | | FeoB1-R | AAGGTGCGGTTCAGGAAGA | |
| *rpoC* | MGR_3807 | RpoC-F | ATCCGTATTTCCATCGCCTCCC | 164 |
| | | RpoC-R | TTGCCGCACAAGCATTCGT | |

**Supplementary Table 4. Strains and plasmids used in this study.**

| Strain and plasmid | Description | Source of reference |
|---|---|---|
| **Strains** | | |
| *M. gryphiswaldense* MSR-1 | Wild type | DSM 6361 |
| F4 | *fur3137*-defective mutant, $Gm^r$ | Ref. 16 |
| F4C | Complement strain of F4, $Gm^r$, $Tc^r$ | Present study |
| F4M | Complement strain of F4 with Fur3137-C9LM14LM16L, $Gm^r$, $Tc^r$ | Present study |
| K15A | Complement strain of F4 with Fur3137-K15A, $Gm^r$, $Tc^r$ | Present study |
| R57A | Complement strain of F4 with Fur3137-R57A, $Gm^r$, $Tc^r$ | Present study |
| H33AH90A | Complement strain of F4 with Fur3137-H33AH90A, $Gm^r$, $Tc^r$ | Present study |
| E108AH125A | Complement strain of F4 with Fur3137-E108AH125A, $Gm^r$, $Tc^r$ | Present study |
| *E. coli* S17-1 | *thiendArecAhsdR* with RP4-2-Tc::Mu-Km::Tn7 integrated in chromosome, $Sm^r$ | Ref. 17 |
| **Plasmid** | | |
| pRK415 | Broad host range cloning vector, $Tc^r$ | Ref. 18 |
| pRKF4M | pRK415 containing *tri-fur3137*, $Tc^r$ | Present study |
| pRKFK15A | pRK415 containing *fur3137*-K15A, $Tc^r$ | Present study |
| pRKFR57A | pRK415 containing *fur3137*-R57A, $Tc^r$ | Present study |
| pRKFH33AH90A | pRK415 containing *fur3137*-H33AH90A, $Tc^r$ | Present study |
| pRKFE108AH125A | pRK415 containing *fur3137*-E108AH125A, $Tc^r$ | Present study |

## Supplementary References


1. Ochsner, U.A. & Vasil, M.L. Gene repression by the ferric uptake regulator in *Pseudomonas aeruginosa*: cycle selection of iron-regulated genes. *Proc. Natl. Acad. Sci. U.S.A.* **93**, 4409-14 (1996).
2. Davies, B.W., Bogard, R.W. & Mekalanos, J.J. Mapping the regulon of *Vibrio cholerae* ferric uptake regulator expands its known network of gene regulation. *Proc. Natl. Acad. Sci. U.S.A.* **108**, 12467-72 (2011).
3. Butcher, J. *et al.* Structure and regulon of *Campylobacter jejuni* ferric uptake regulator Fur define apo-Fur regulation. *Proc. Natl. Acad. Sci. U.S.A.* **109**, 10047-52 (2012).
4. Baichoo, N. & Helmann, J.D. Recognition of DNA by Fur: a reinterpretation of the Fur box consensus sequence. *J. Bacteriol.* **184**, 5826-32 (2002).
5. Rohs, R. *et al.* The role of DNA shape in protein-DNA recognition. *Nature* **461**, 1248-53 (2009).
6. West, S.M., Rohs, R., Mann, R.S. & Honig, B. Electrostatic interactions between arginines and the minor groove in the nucleosome. *J. Biomol. Struct. Dyn.* **27**, 861-6 (2010).
7. Kitayner, M. *et al.* Diversity in DNA recognition by p53 revealed by crystal structures with Hoogsteen base pairs. *Nat. Struct. Mol. Biol.* **17**, 423-9 (2010).
8. Chen, Y. *et al.* DNA binding by GATA transcription factor suggests mechanisms of DNA looping and long-range gene regulation. *Cell Rep.* **2**, 1197-206 (2012).
9. Eldar, A. *et al.* Structural studies of p53 inactivation by DNA-contact mutations and its rescue by suppressor mutations via alternative protein-DNA interactions. *Nucleic Acids Res.* **41**, 8748-59 (2013).
10. Chang, Y.P. *et al.* Mechanism of origin DNA recognition and assembly of an initiator-helicase complex by SV40 large tumor antigen. *Cell Rep.* **3**, 1117-27 (2013).
11. Meijsing, S.H. *et al.* DNA binding site sequence directs glucocorticoid receptor structure and activity. *Science* **324**, 407-10 (2009).
12. Tsodikov, O.V. & Biswas, T. Structural and thermodynamic signatures of DNA recognition by *Mycobacterium tuberculosis* DnaA. *J. Mol. Biol.* **410**, 461-76 (2011).
13. Xu, Y. *et al.* Tet3 CXXC domain and dioxygenase activity cooperatively regulate key genes for Xenopus eye and neural development. *Cell* **151**, 1200-13 (2012).
14. de Lorenzo, V., Wee, S., Herrero, M. & Neilands, J.B. Operator sequences of the aerobactin operon of plasmid ColV-K30 binding the ferric uptake regulation (fur) repressor. *J. Bacteriol.* **169**, 2624-30 (1987).
15. Escolar, L., Perez-Martin, J. & de Lorenzo, V. Binding of the fur (ferric uptake regulator) repressor of *Escherichia coli* to arrays of the GATAAT sequence. *J. Mol. Biol.* **283**, 537-47 (1998).
16. Qi, L. *et al.* Fur in *Magnetospirillum gryphiswaldense* influences magnetosomes formation and directly regulates the genes involved in iron and oxygen metabolism. *PLoS ONE* **7**, e29572 (2012).
17. Simon, R., Priefer, U. & Puhler, A. A Broad Host Range Mobilization System for In Vivo Genetic Engineering: Transposon Mutagenesis in Gram Negative Bacteria. *Nat. Biotech.* **1**, 784-791 (1983).
18. Scott, H.N., Laible, P.D. & Hanson, D.K. Sequences of versatile broad-host-range vectors of the

RK2 family. *Plasmid* **50**, 74-9 (2003).
